# The archaeal triphosphate tunnel metalloenzyme *Sa*TTM defines structural determinants for the diverse activities in the CYTH protein family

**DOI:** 10.1016/j.jbc.2021.100820

**Published:** 2021-05-23

**Authors:** Marian S. Vogt, Roi R. Ngouoko Nguepbeu, Michael K.F. Mohr, Sonja-Verena Albers, Lars-Oliver Essen, Ankan Banerjee

**Affiliations:** 1 Department of Chemistry, Philipps-Universität Marburg, Marburg, Germany; 2 Institute of Pharmaceutical Sciences, Albert-Ludwigs-Universität Freiburg, Freiburg, Germany; 3 Institute of Biology II, Molecular Biology of Archaea, Albert-Ludwigs-Universität Freiburg, Freiburg, Germany; 4 Center for Synthetic Microbiology, Philipps-Universität Marburg, Marburg, Germany; 5 Department of Genetics, Philipps-Universität Marburg, Marburg, Germany

**Keywords:** CYTH enzymes, triphosphatase tunnel metalloenzyme, two-metal ion mechanism, sequence similarity network, protein structure evolution, AC-IV, CyaB-like class IV adenylyl cyclase, CI, confidence interval, CYTH, CyaB-thiamine triphosphatase, IMAC, immobilized metal affinity chromatography, Pi, orthophosphate, PPi, pyrophosphate, PPPi, triphosphate, SSN, sequence similarity network, ThTPase, thiamine triphosphatase, TTM, triphosphate tunnel metalloenzyme

## Abstract

CYTH proteins make up a large superfamily that is conserved in all three domains of life. These enzymes have a triphosphate tunnel metalloenzyme (TTM) fold, which typically results in phosphatase functions, *e.g.*, RNA triphosphatase, inorganic polyphosphatase, or thiamine triphosphatase. Some CYTH orthologs cyclize nucleotide triphosphates to 3′,5′-cyclic nucleotides. So far, archaeal CYTH proteins have been annotated as adenylyl cyclases, although experimental evidence to support these annotations is lacking. To address this gap, we characterized a CYTH ortholog, *Sa*TTM, from the crenarchaeote *Sulfolobus acidocaldarius*. Our *in silico* studies derived ten major subclasses within the CYTH family implying a close relationship between these archaeal CYTH enzymes and class IV adenylyl cyclases. However, initial biochemical characterization reveals inability of *Sa*TTM to produce any cyclic nucleotides. Instead, our structural and functional analyses show a classical TTM behavior, *i.e.*, triphosphatase activity, where pyrophosphate causes product inhibition. The Ca^2+^-inhibited Michaelis complex indicates a two-metal-ion reaction mechanism analogous to other TTMs. Cocrystal structures of *Sa*TTM further reveal conformational dynamics in *Sa*TTM that suggest feedback inhibition in TTMs due to tunnel closure in the product state. These structural insights combined with further sequence similarity network–based *in silico* analyses provide a firm molecular basis for distinguishing CYTH orthologs with phosphatase activities from class IV adenylyl cyclases.

Bacterial CyaB-like class IV adenylyl cyclases (AC-IVs) and mammalian thiamine triphosphatases (ThTPases) are founding members of the CYTH-like domain superfamily (1). CYTH enzymes are found in all three domains of life and can be traced back to the last common ancestor, which makes them appealing targets for studying protein evolution (1). Besides CyaB and ThTPases, the CYTH-like domain superfamily includes inorganic triphosphatases (2, 3), which together are wrapped up in a CYTH domain subfamily, and Cet1-like RNA-triphosphatases that have their own subfamily of mRNA triphosphatase Cet1-like enzymes (4).

The common feature of CYTH enzymes is an eight-stranded β-barrel tunnel architecture, termed the triphosphate tunnel metalloenzyme (TTM) fold, which was first found in the crystal structure of the fungal Cet1 RNA triphosphatase (4, 5). The hallmark of CYTH enzymes is an N-terminal *ExExK* motif that is involved in metal ion coordination and substrate binding (3). The opposing acidic and basic patches shape the substrate binding tunnel. Although the overall coordination of the triphosphate itself is very similar among CYTH enzymes, the directionality of asymmetric organic substrates such as triphosphate nucleotides or thiamine triphosphate within the tunnel determines catalytic specificity (3). For triphosphatases and ThTPases, the organic moiety points toward a C-terminal helix, termed plug helix (3, 6). Such enzymes catalyze the hydrolysis of the triphosphate by a two-metal ion mechanism using metal one to establish the catalytically competent binding of the triphosphate moiety and metal two to position and activate a water for nucleophilic attack onto the γ-phosphate moiety (3). Accordingly, triphosphatases generate pyrophosphate and an orthophosphate by hydrolysis of the β–γ phosphoanhydride bond. The situation for AC-IV enzymes in the CYTH superfamily is different. Here, the directionality of the organic moiety binding within the tunnel fold is inverted causing a reversed placement of the α- and γ-phosphates within the binding tunnel (7). By comparing AC-IV with already described AC classes, a general two-metal ion mechanism was suggested for the cyclization of ATP to 3′,5′-cAMP using the second metal to lower the activation energy (7, 8, 9, 10).

The founding members of the CYTH superfamily are involved either in mammalian thiamine triphosphate metabolism or bacterial signal transduction *via* 3′,5′-cyclic nucleotides. Some members of the CYTH superfamily are parts of multidomain proteins by being fused to HD hydrolase, exopolyphosphatase, nucleotidyl kinase, or CHAD domains (1). It was postulated that CYTH orthologs play a central role at the interface between nucleotide and phosphate metabolism (1, 11). Archaeal representatives encode at least one copy of a CYTH ortholog in their genomes, which are generally annotated as adenylyl cyclases. 3′,5′-Cyclic AMP has been described for only a few archaeal species with a role in starvation and cell cycle (12, 13). In a search for a potential nucleotidyl cyclase in the crenarchaeote *Sulfolobus acidocaldarius,* we identified *Saci_0718,* a member of the CYTH superfamily and annotated as CyaB-like class IV adenylyl cyclase. Although the substrate binding mode and the hydrolysis mechanisms differ between triphosphatases and CyaB (3), at the sequence level, *Saci_*0718 shares ∼29% identity with the *Yersinia pestis* cyclase CyaB and with the inorganic triphosphatase from *Clostridium thermocellum*.

Here, we have performed a structure–function analysis of *Saci*_0718 and designated it as *Sa*TTM due to its triphosphatase activity. Its structures in apo and substrate-bound (triphosphate and ATP) states revealed transition between open and closed states during catalysis. Trapping of the Michaelis complex in its calcium-inhibited state confirmed a two-metal ion mechanism for *Sa*TTM. Furthermore, this archaeal TTM is subject to feedback inhibition by its reaction product pyrophosphate. Our comparative structural and bioinformatic analysis of CYTH allowed us to identify molecular determinants, which clearly discriminate the adenylyl cyclase from the different phosphatase activities. This provides now an improved rationale for correct functional annotation within the broad CYTH domain family.

## Results

### Archaeal CYTH proteins are functionally diverged from CyaB-like class IV adenylyl cyclases

To date, the CYTH-like domain superfamily (IPR033469) comprises 41,000 enzymes from all three domains of life divided into two subfamilies, the smaller mRNA triphosphatase Cet1-like(IPR004206) and the bigger subfamily of CYTH domain proteins (IPR023577) including adenylyl cyclases, ThTPases, and inorganic tri- or polyphosphatases (also known as triphosphatase tunnel metalloenzymes or TTMs). The discrimination between Cet1-like and CYTH enzymes is also consistent within the Pfam database (mRNA_triPase*:* PF02940 and CYTH*:* PF01928). Hence, we aimed to obtain a detailed understanding of the CYTH family by combining biochemical analysis combined with structural snapshots of the catalytic mechanism, as the functional annotations of CYTH enzymes are still vague and misleading among TTMs and adenylyl cyclases.

**Figure 1:**
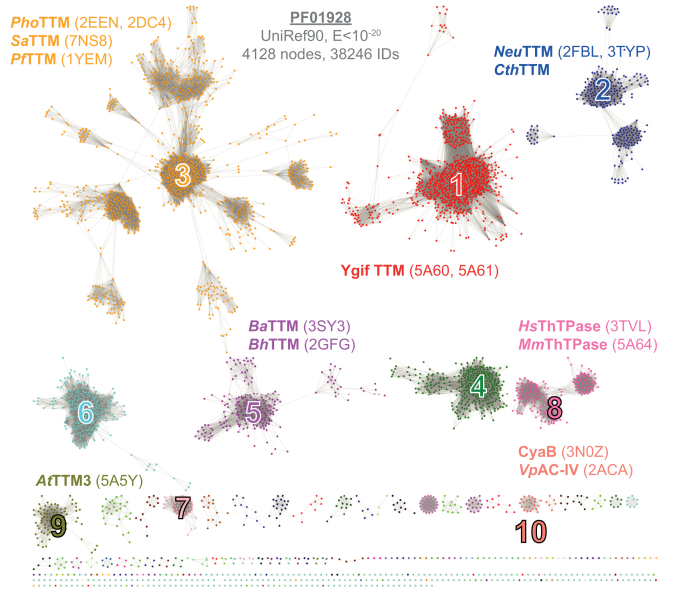
**Sequence similarity network of CYTH proteins**. The CYTH family PF01928 is presented as a sequence-similarity network, generated with the EFI-EST using the domain boundaries and a final alignment score of 10^−20^ (14). Each node represents sequences with >40% identity, and the final network was subjected to the cluster analysis implemented by the EFI-EST. Accordingly, the ten largest clusters were subjected for further analysis, including all currently described CYTH enzymes (see Table S1). Known TTMs are found in clusters 1 to 3, 5, and 9. ThTPase enzymes appear exclusively in cluster 8 and CyaB/AC-IVs belong to cluster 10. Proteins from cluster 4 are unknown, and cluster 7 members lack the hallmark motif *ExExK*. If available, Protein Data Bank entries are given in parentheses. The network with E<10^−15^ and WEBLOGOs of clusters 1 to 10 are shown in Figures S7 and S8.

To ensure unambiguous assignment, we chose the Pfam family CYTH (PF01928) as the least common denominator for our analysis. As stated above, this protein family contains enzymes with known functions as TTMs, AC-IVs, and ThTPases. We therefore performed a systematic sequence similarity network (SSN) analysis of the CYTH superfamily PF01928 using EFI-EST (14). The network generated with an E-value of 10^−20^ against the CYTH-domain boundaries for 38,246 sequences clustered to 4128 nodes with pairwise sequence identities of >40% within the nodes, which are connected by a total of 121,791 edges. This SSN analysis covering all members of the CYTH enzymes of PF01928 showed 10 major distinct clusters (Fig. 1). Among these clusters, three of them (clusters 1, 4, and 7) comprise members adopting a multidomain architecture, while the length histograms for the remaining seven clusters are in the expected range of ∼160 to 200 amino acids for single-domain proteins (Fig. S7*A*). For example, cluster 1 contains YgiF from *Escherichia coli*, which possesses a two-domain architecture with an N-terminal CYTH and a C-terminal CHAD domain (PF05235) (3). Similar modular architectures including the CHAD domain can be found in cluster 4, which does not contain any structurally described homologs and whose members occur mostly in actinobacteria. The additional domain in cluster 7, whose members are exclusively found in *Viridiplantae* and *Amoebozoa*, is a phosphoribulokinase domain (PF00485).

Of interest, the SSN analysis discriminates the cyaB-like class IV adenylyl cyclase (7, 15) (cluster 10) from other triphosphatases. Cluster 10 is a relatively small cluster within the SSN by comprising only 141 orthologs of bacterial origin. The active site structure and a two-metal ion mechanism of *Y. pestis* AC-IV was proposed previously (10). Like AC-IV, the ThTPases form a separate cluster, cluster 8, that is distinct from other triphosphatases. The key molecular determinant of ThTPase was reported previously (3, 6). The enzymes described as inorganic triphosphatases are distributed among different clusters. *Neu*TTM and *Cth*TTM were found in cluster 2; TTM3 from *Arabidopsis thaliana* is a member of cluster 9 (3, 16, 17). The two other described enzymes from *A. thaliana* (TTM1 and TTM2) are in cluster 7, and interestingly they utilize pyrophosphate and not triphosphate (18). *At*TTM1 and *At*TTM2 do not possess the TTM signature motifs required for catalysis (Table S1). In cluster 5, unpublished structures annotated before as adenylyl cyclase from *Bacillus anthracis* and *Bacillus halodurans* can be found. A member of this cluster from *Staphylococcus aureus* has further been described to be incompetent in cAMP formation and to be distinct from class IV adenylyl cyclases (19). Cluster 3 contains orthologs of mostly archaeal origin from *Pyrococcus furiosus* and *Pyrococcus horikoshii,* as well as *Saci_*0718 (Uniprot: *Q4JAT2*). The SSN analysis clearly indicates that archaeal CYTH enzymes are functionally diverge from CyaB-like class IV adenylyl cyclases.

### *S. acidocaldarius* CYTH enzyme is a triphosphate tunnel metalloenzyme

**Figure 2:**
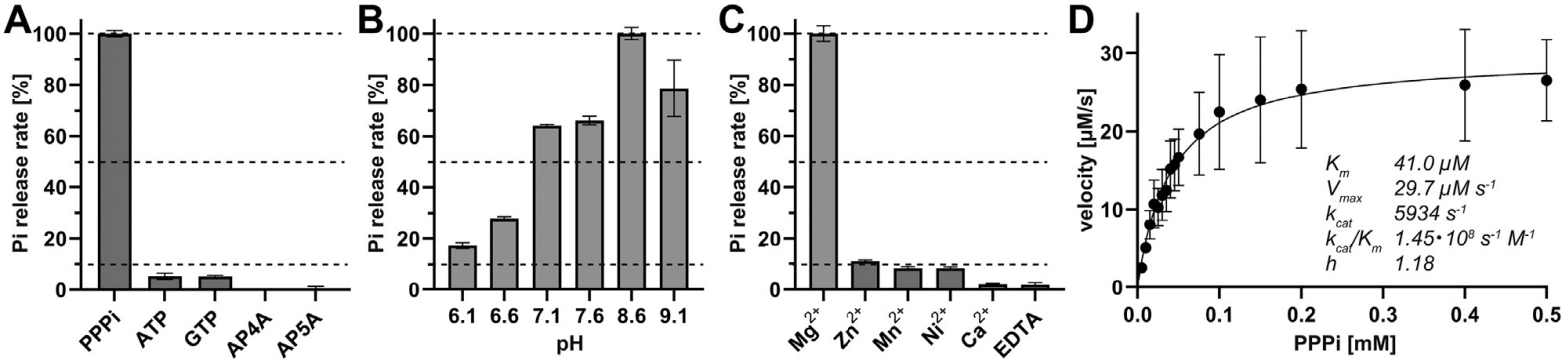
***Sa*TTM prefers inorganic triphosphate over nucleotides.***A*, for substrate specificity, 5 nM *Sa*TTM was incubated with 2 mM MgCl_2_ and 0.1 mM substrate at pH 8.6 and 75 °C for 10 min. *B*, the pH optimum of *Sa*TTM peaks at pH 8.6 within the tested range of 6.1 to 9.1, using the conditions from (*A*). *C*, different metal ions (Mg^2+^, Zn^2+^, Mn^2+^, Ni^2+^, and Ca^2+^, EDTA as control, 2 mM each) were examined to show their effect on *Sa*TTM activity, using the conditions from (*A*) and an incubation time of 5 min. *D*, v/S diagram of *Sa*TTM using 5 nM enzyme, 0.005 to 0.5 mM PPPi as substrate at pH 8.6 and 75 °C. Nonlinear fitting for the kinetic parameter *K*_m_ (41.0 μM) resulted in a 95% confidence interval (CI) of 29.8 to 55.4 μM, for *V*_max_ (29.7 μM s^−1^) a 95% CI of 26.7 to 33.0 μM s^−1^, and for the Hill coefficient (1.18) a 95% CI of 0.79 to 1.69, respectively.

To gain in-depth biochemical and structural insights into this protein, we heterologously overproduced a C-terminal His_6_-tagged variant of *Saci*_0718 in *E. coli.* The recombinant protein was purified to homogeneity using an initial heat denaturation step to remove *E. coli* proteins followed by IMAC and subsequent size exclusion chromatography as a polishing step. The enzyme formed a dimer in solution corresponding to 56 kDa compared with standards (Fig. S1). Dimerization behavior is typical among other CYTH proteins (11). The first ambiguity to be resolved was determination of its substrate preference and the nature of its hydrolysis products. Initially, we tested its activity against ATP using Mg^2+^ as cofactor at 75 °C and the reaction product analyzed by HPLC (Fig. S2*A*). This experiment clearly showed that the enzyme is capable of hydrolyzing ATP to ADP and Pi without any formation of cAMP. *Saci*_0718 showed no base specificity during its phosphohydrolase reaction and able to utilize both ATP or GTP with similar efficiency (Fig. 2*A*). As the reaction products are diphosphate nucleosides and not (c)AMP/GMP, we used nonhydrolyzable analogs of ATP, AMPCPP, and AMPPCP to identify the nucleophilic attack site (Fig. S2*B*). It shows that Saci_0718 acts on the terminal phosphate of ATP, as the enzyme was active on AMPCPP but no phosphate release was observed for AMPPCP.

Next, we tested triphosphate (PPPi) as substrate. Under the conditions outlined before *Saci_*0718 is ∼19 times more active compared with NTP as substrate. The result is consistent with the previous reports of tunnel metalloenzymes (2, 3). We also observed that the enzyme is able to hydrolyze higher polyphosphates like tetraphosphate with final formation of pyrophosphate and phosphate (Fig. S3). As triphosphate seems to be the preferred substrate, we named *Saci_0718 Sa*TTM (*S. acidocaldarius* triphosphate tunnel metalloenzyme). The optimal assay conditions were observed at a basic pH 8.6 at 75 °C using Mg^2+^ as a cofactor, whereas Zn^2+^, Mn^2+^, and Ni^2+^ could be used as weaker cofactor alternatives. Similar to the metal ion chelator EDTA, calcium exerted an inhibitory effect on phosphatase activity (Fig. 2, *B* and *C*). At optimal conditions, kinetic measurement revealed a classical Michaelis–Menten behavior with a *K*_*m*_ of 41.0 μM and a *V*_*max*_ of 29.7 μM s^−1^ (Fig. 2*D*). This shows a highly efficient catalysis performed by *Sa*TTM with a *k*_cat_/*K*_*m*_ of 1.45 × 10^8^ s^−1^ M^−1^ close to the diffusion limit and a turnover rate, *k*_cat_, of 5934 s^–1^, which is in the range of untagged *Neu*TTM (7900 s^−1^) (16).

### Crystal structure of the *Sa*TTM dimer

**Figure 3:**
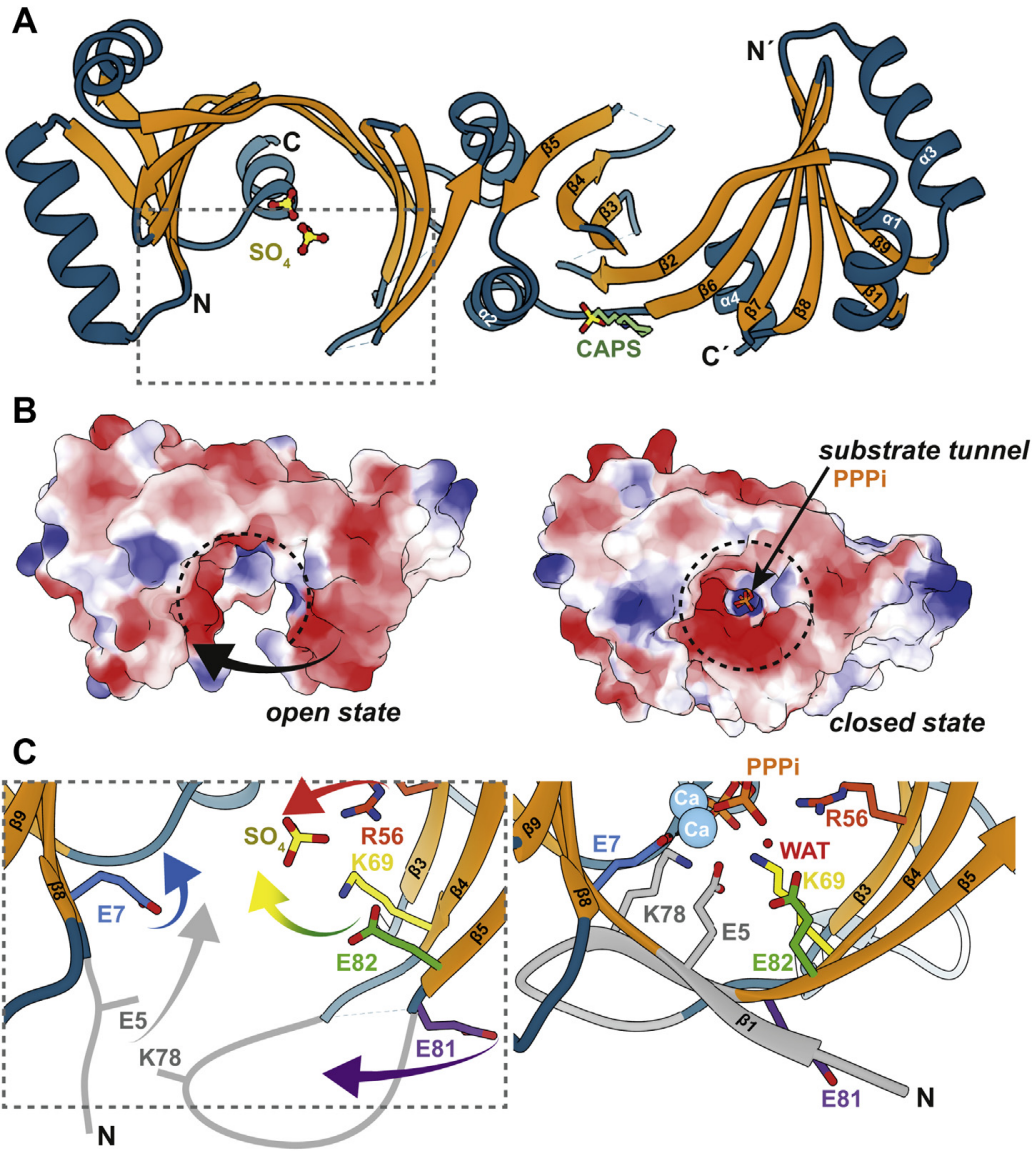
***Sa*TTM dimers transition between open and closed states**. *A*, the open homodimer of *Sa*TTM is shown as cartoon with the beta barrel being highlighted in *orange* and the rest being colored in *petrol*. The secondary structure elements are indicated on the right protomer. The cocrystallized sulfates and CAPS molecules (sticks with *green* carbons, *red* oxygens, and *yellow* sulfurs) are shown in one of the protomers for clarity, but are present in both, as the dimer has been generated by the symmetry mate due to just one molecule per AU. N and C termini of the protomers are indicated, the *dashed lines* indicate missing electron density for residues P44–R49 and Q73–R80. *B*, the *left panel* shows the top orientation of the open states' surface as indicated by the *arrow* using the electrostatic coloring from negative (*red*) to positive (*blue*). The *arrow* indicates the closing movement upon ligand binding, resulting in the closed conformation shown on the *right* with the PPPi (*orange* and *red sticks*) substrate in *orange* bound within the tunnel. The *dashed (open) circles* indicate the conformation switch. *C*, conformational changes of *Sa*TTM accompanying open-to-closed state transition. Of note, the disordered N terminus (M1–E5) and loop Q73–R80 (open state: schematically depicted in *gray*) become ordered in the closed state.

For structural information, we crystallized *Sa*TTM in different catalytically relevant complexes. Initial attempts to produce diffraction quality crystals using apoenzyme were unsuccessful, but crystallization was finally possible under a high-sulfate condition. In contrast, ligand-bound states regularly produced high hit numbers for diffraction quality crystals, indicating that *Sa*TTM is highly flexible in solution and stabilized upon ligand binding. Crystals of *Sa*TTM in its different catalytic states were obtained after a week at 4 °C that diffracted to 1.75- to 2.3-Å resolution. For structure determination, the *P. horikoshii* OT3 *PH1819* (Protein Data Bank [PDB] entry 2EEN, 39% identity with *Sa*TTM)-based homology model of *Sa*TTM was used as a search model in molecular replacement. *Sa*TTM was crystallized as an open-state homodimer (Fig. 3*A*), bound with two sulfate anions instead of the ATP that was included in cocrystallization. Of note, a solution structure of mouse thiamine triphosphatase (PDB 2JMU) reported a similarly open state. The open state of *Sa*TTM was crystallized in space group *P*4_1_2_1_2 with one monomer per asymmetric unit. The polypeptide is defined by electron density for R6-A176 but lacks completely residues P44-R49 and Q73-R80 due to missing density. The general topology of *Sa*TTM is described by an eight-stranded β-barrel comprising the antiparallel strands β1-β9/β5-β4-β3-β2-β6-β7-β8(-β1) with three interspersing α helices and the C-terminal plug helix α4. The dimerization of *Sa*TTM was also found in the crystal structure formed by two symmetry mates along the α2 helices and parts of the preceding β5 strand. PDBePISA revealed that the dimer interface covered 875 Å^2^ with a CSS score of 0.32 and accounts for 8.5% of the total solvent-accessible area of one protomer (20). The dimerization is mainly established by hydrophobic interactions of residues from β3, β4, β5, and α2 and salt bridges between K99 from one protomer and E81 from the other protomer. The C-terminal α4 plug helix blocks the tunnel entry from one side. It was previously reported that this plug helix plays a major role in determining substrate preference (17).

### Catalytic ensemble of *Sa*TTM

Next, we aimed to trap the Michaelis complex of *Sa*TTM, for which we cocrystallized the enzyme together with PPPi or ATP and the respective cofactors Mg^2+^ or Ca^2+^ at 4 °C. Substrate-bound states of *Sa*TTM crystallized in a closed tunnel conformation, highlighting the structural rearrangement upon ligand binding (Fig. 3*B*). This supports the previous notion that CYTH enzymes are highly flexible and can breathe during their catalytic cycle (6, 17). In all states but the open state, a closed tunnel conformation including the missing stretches P44-R49 (loop β2/β3) and Q73-R80 (loop β4/β5) of the open state were observed (Fig. 3*C*).

**Figure 4:**
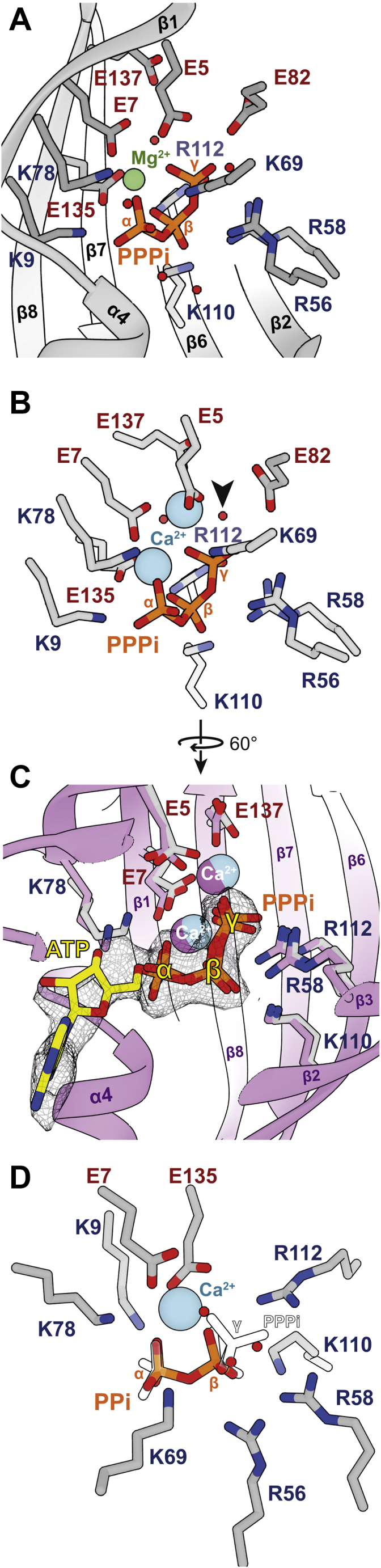
**Catalytic snapshots of *Sa*TTM**. *A*, residues (*gray*) in the binding tunnel of *Sa*TTM are shown coordinating its substrate PPPi (*orange sticks*) and a magnesium ion (*green sphere*) at the metal-binding site 1. *B*, same orientation as in A of residues (*gray*) binding PPPi and two calcium ions (*blue sphere*) is shown. The attacking water molecule is marked by an *arrowhead*. *C*, for identification of triphosphate orientation, *Sa*TTM is cocrystallized with ATP (*yellow*) and two calcium ions in the context of secondary structure elements (as indicated) and residues in *purple*. The superposition with the PPPi-calcium state from (*B*) is shown with the indicated residues to assign the α- and γ-positions of the symmetric substrate in *A*, *B*, and *D*. *D*, the product-bound state of *Sa*TTM in complex with PPi and calcium is shown with the same coloring scheme as in (*B*). The PPPi is shown with contours only, identifying the α- and β-positions and further show that the three indicated waters (*red spheres*) localize at the γ-phosphate oxygen positions.

As shown for *At*TTM3 (3), we observed electron density in the PPPi⋅Mg^2+^ state for just one metal ion octahedrally coordinated by PPPi, E7, and E135 (Fig. 4*A* and S4*A*), whereas the second ion necessary for water activation was missing (Fig. S5*C*). When using a complete PPPi molecule during refinements, we found higher B-factors for the γ-phosphate. Therefore, the molecule was split into PPi and γ-Pi and occupancies were separately refined. This revealed that only 76% of the substrate-binding site were occupied with γ-phosphates, suggesting a slow turnover of the triphosphate *in crystallo*.

**Figure 5:**
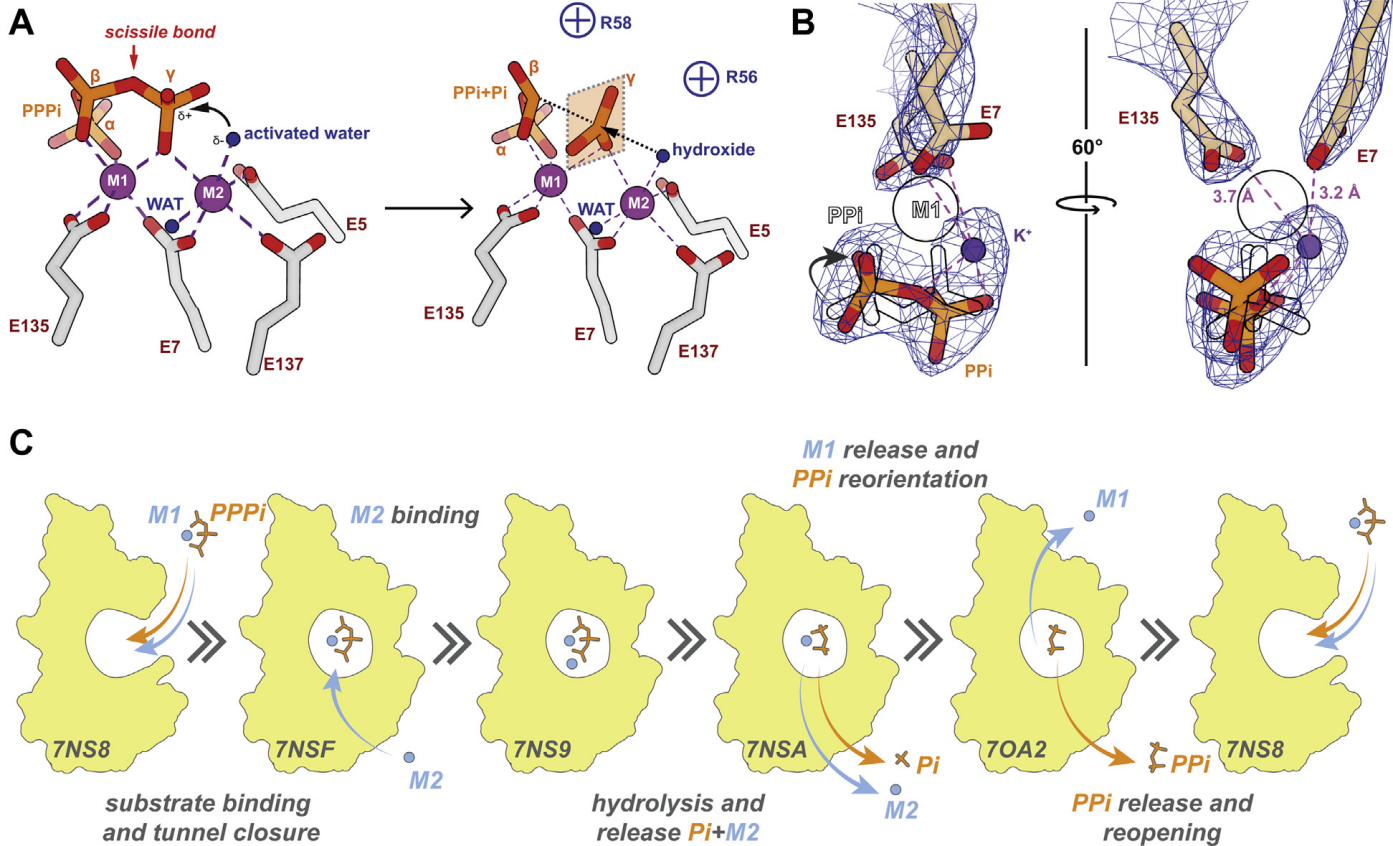
**Two-metal ion catalysis by TTMs**. *A*, in two-metal ion catalysis metal ion 2 (M2) activates a coordinated water for inline attack on the γ-phosphate with αβ-pyrophosphate as leaving group. Of note, deprotonation of this water does not involve side chains acting as general base. Metal ion 1 (M1) serves for charge neutralization and binding of the αβ-pyrophosphate moiety. Apart from R56 and R58 other basic residues stabilizing the triphosphate group include K69, K110, and R112. *B*, the 2.7-Å crystal structure of the PPi⋅Mg^2+^ complex shows a lack of coordinated Mg^2+^ ions. The pyrophosphate group adopts a different conformation than in the PPi⋅Ca^2+^ and PPPi complexes (shown in *silhouette* and indicated by *arrow*) and recruits a potassium ion (*purple*). The position of M1 is shown as *black circle*. *C*, catalytic cycle of TTM as mapped by SaTTM complex structures (Protein Data Bank IDs on bottom left of the respective state). In this model, binding of Mg^2+^-complexed substrate induces open –close state transition of the TTM and ordered release of metal ions M2 and M1 after hydrolysis. The loss of M1 alters the conformation of the pyrophosphate and triggers tunnel back opening.

The PPPi⋅Ca^2+^ state (Figs. 4*B* and S5*D*) revealed not only metal ion 1 (M1) but also the water-activating position of metal ion 2 site (M2) present in the electron density. The two Ca^2+^ ions are coordinated by PPPi, E5, E7, and E137 (Fig. S4*B*), thereby positioning a water molecule in an inline-attack position 3.2 Å away from the γ-phosphate phosphorous as observed for the *E. coli* YgiF⋅PPPi states (PDB: 5A60, 5A61). Here, no differences of γ-phosphate B-factors and occupancies were observed, indicating no hydrolysis during crystallization, which is in line with activity assays in the presence of Ca^2+^ (Fig. 2*C*). As *Sa*TTM catalyzes asymmetrical cleavage of a symmetric substrate it is difficult to judge the directionality of the substrate, a prerequisite for firm mechanistic conclusions. Hence, we solved the ATP-bound form of *Sa*TTM in the presence of Ca^2+^ ions (Figs. 4*C* and S5*B*). The *Sa*TTM⋅ATP state revealed the adenosine base on the side of the plug helix α4, defining the orientation of the triphosphate moiety. It also showed that the catalytic mechanism of *Sa*TTM follows a classical inorganic triphosphatase, but not an AC-IV, providing the structural base of TTM function. Of interest, the predicted inline attack by an activated water nucleophile (see Figs. 4*B*, 5*A*, and S4*B*) onto the γ-phosphate apparently misses a general-base support by protein side chains and is consistent with the high pH optimum of 8.6 for *Sa*TTM. Upon the structural and biochemical confirmation of *Sa*TTM's inability to use calcium as reaction-competent cofactor we wondered how Ca^2+^ competes with Mg^2+^. Hence, we performed a titration experiment with increasing concentrations of calcium on a magnesium-catalyzed reaction (Fig. S6*A*). As expected, we observed at equimolar concentrations a highly reduced activity of *Sa*TTM using PPPi as substrate, and almost no turnover with 5-fold excess of the inhibiting metal ion, indicating that calcium is a potent inhibitor of TTM.

For obtaining structural snapshots of the product state, we determined crystal structures of *Sa*TTM in complex with pyrophosphate (Figs. 4*D* and 5*B*). Like the Michaelis complex, the postcatalytic state adopts the closed tunnel conformation. By using the superimposed triphosphate state, the two phosphates can be assigned in the PPi⋅Ca^2+^ state to the α- and β-positions complexing the calcium ion at metal 1 position (Figs. 4*D* and S5*A*). Three water molecules occupy the positions corresponding to the oxygens of the substrate's γ-phosphate moiety. The product-bound states clearly suggest an inhibitory effect of pyrophosphate on TTM activity due to tunnel closure. To confirm this notion, we performed another titration-inhibition experiment (Fig. S6*B*). The activity of *Sa*TTM was rapidly decreased and already halved when using a ten-thousandth of the substrate concentration. Although this effect plateaus until equimolar concentrations, the inhibition upon 10-fold excess is amplified and with 100-fold excess the enzyme activity was completely abolished.

The product inhibition of *Sa*TTM by pyrophosphate in the closed state suggests that product release may be a rate-limiting step and that the full catalytic cycle of TTMs (Fig. 5*C*) requires fluctuation between substrate-accessible open states and catalytically active closed states. Of interest, the PPi⋅Mg^2+^ state shows release of the divalent ion M1 and a conformational change for the bound pyrophosphate group (Fig. 5*B* and S5*E*). Calcium-mediated inhibition of TTM may hence derive not only from the different coordination of the γ-phosphate and the polarization of the attacking water but also from a stabilization of the pyrophosphate-bound product state, which blocks subsequent tunnel opening.

### Role of the ordered water molecule in substrate binding and catalysis

**Figure 6:**
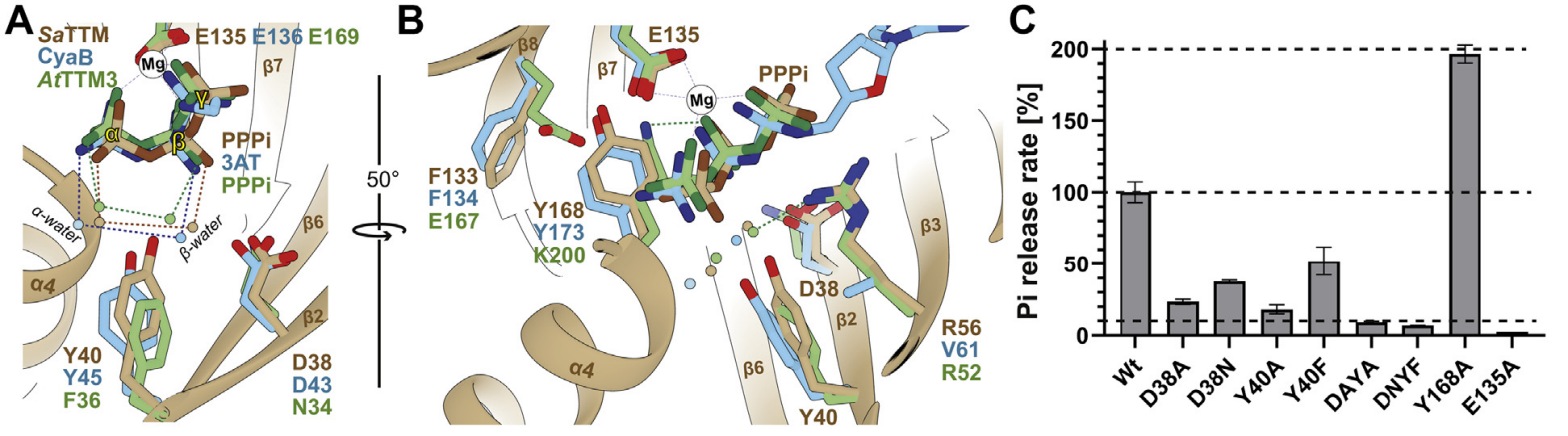
**Different modes of substrate coordination among CYTH enzymes utilizing two conserved waters**. *A* and *B*, superposition of substrate binding mode of *Yp*CyaB (3N0Z, *blue*), *At*TTM3 (5A5Y, *green*), and *Sa*TTM (7NS8, *brown*) is shown in the context of secondary structure elements of *Sa*TTM from two different angles as indicated. The magnesium cofactor (*white sphere*) from *Sa*TTM is shown. Although mechanistically different, the binding modes of *Yp*CyaB and *Sa*TTM are highly similar regarding the establishment of the substrate waters, whereas *At*TTM3 differs strikingly. *C*, PPPi hydrolysis activities (substrate concentration 100 μM) of the indicated residues show the impact of correct water placement by the *DxY* motif and a positive effect on activity of a loosened plug helix.

During SSN analysis we found at a less stringent alignment score of 10^−15^ that CYTH enzyme orthologs from cluster 3 (including *Sa*TTM) and cluster 10 (including CyaB) belong to the same parental cluster (cluster 3 in Fig. S7*B*). This suggests generally higher sequence and structural similarity between CyaB and *Sa*TTM-like enzymes compared with other TTM subfamilies, while the triphosphatase-like catalytic mechanism of *Sa*TTM is apparently shared with CYTH enzyme orthologs already well separated at less stringent thresholds. To find a structural rationalization for this phenomenon, we compared substrate-bound states of *Yp*CyaB and *Sa*TTM from the same parental cluster with *At*TTM3 from cluster 9 (Fig. 6, *A* and *B*). We identified two water molecules involved in substrate coordination and present in all structures of ligand-bound CYTH superfamily proteins. These waters are located opposite the metal ions and directly hydrogen bonded to the α- and β-phosphates, hence termed as α- and β-water (Fig. 6*A*). Although β-water in *Sa*TTM and *Yp*CyaB made a bidentate contact with the *DxY* motif, this interaction is absent in *At*TTM3 owing to a slightly moved water and an *NxF* motif. Here, the asparagine is 4 Å away and the phenylalanine lacks the ability to establish a hydrogen bond. This β-water establishes a hydrogen bond with R52 on β3, explaining the well-ordered β-water in *At*TTM3 (Fig. 6*B*). The deviations in substrate coordination can also be observed on the metal ion–binding side. Although *Sa*TTM and *Yp*CyaB share the same arrangement by shaping the binding pocket in close proximity to the α-phosphate using a combination of two conserved aromatic residues (Y168/173 and F133/134), the situation in *At*TTM3 is different. Here, a salt bridge ensemble of E167 and K200 is used for direct electrostatic interaction with the γ-phosphate. As we observed this highly similar binding mode of α- and β-phosphates in *Sa*TTM and *Yp*CyaB, we wondered about the impact of these residues on enzyme activity. Hence, we mutated the DxY motif and the central tyrosine, Y168, as well as E135 as a nonactive control owing to its central role in M1 binding (Fig. 6*C*). As hypothesized, the *Sa*TTM D38A and Y40A mutants showed reduced activity compared with the wildtype. Intuitively, less impact on the catalysis was observed for the conservative D38N and Y40F mutants compared with the alanine mutants, as similar hydrogen bond formation is still possible in the asparagine variant and in the phenylalanine variant we still have the aspartate for β-water coordination. The double mutant had an accumulating effect with less than 10% of wildtype activity, strengthening the importance of the *DxY* motif in coordinating properly the water–substrate complex for catalysis. Of interest, the Y168A variant showed a 2-fold turnover rate compared with the wildtype enzyme, whereas the corresponding K200A mutant in the *At*TTM3 mutant showed reduced activity (3). These residues are part of the plug helix, which occludes the tunnel opening. This structural element plays a critical role in substrate preference and enzyme activity (17). Y168 interacts with F133 from β5, holding the plug helix in the observed conformation, while not playing a role in direct electrostatic interaction with the triphosphate substrate as known from K200 in *At*TTM3 (Fig. 6*C*). By mutating the tyrosine, the flexibility of the plug helix was increased and tunnel back opening was favored, which results in an enhanced activity of the *Sa*TTM^Y168A^ variant.

## Discussion

### Structural signature that defines versatile CYTH function

**Figure 7:**
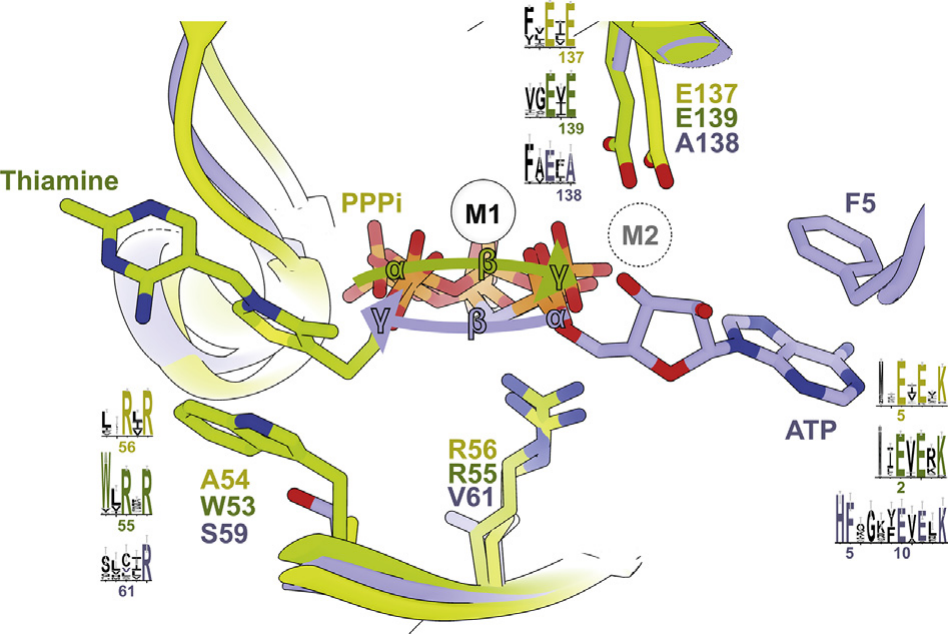
**Key motifs for substrate orientation and specificity of PF01928 enzymes**. Superposition of *Yp*CyaB (3N0Y, *blue*), mouse ThTPase (5A64, *green*), and *Sa*TTM (7NS9, *yellow*) is shown. AC-IV enzymes possess an exclusive, N-terminal *HF* motif for nucleobase stacking interaction by the phenylalanine at the opposite site of the tunnel compared with other triphosphatases. This motif is located four residues before the CYTH hallmark motif *ExExK*. The second motif (*WxRxR*) identifies the ThTPases, where tryptophan (W53) specifically stacks the thiazolium ring of the thiamine triphosphate. TTMs and AC-IV have small residue substitutes and the cyclases further exchanged the otherwise conserved central arginine to a small, neutral residue, which does not sterically hinder correct ribose placement. The third, acidic *ExE* motif is responsible for correct metal coordination. The first glutamate is conserved throughout the whole family, as it is the central residue for metal 1 (M1) coordination, while the latter glutamate establishes the interaction to metal 2 (M2), which is necessary for the catalytic mechanism in TTMs and ThTPases. This glutamate is strictly replaced by an alanine in all AC-IV proteins.

Decades of study on CYTH-containing proteins from the protein family PF01978 established the catalytic mechanisms. However, owing to high sequence similarities between different isofunctional subclusters it proved hard to assign a correct functional annotation to uncharacterized family members. We aimed to integrate our newly gained structural insights with our findings from the SSN and already known CYTH enzymes to establish a detailed understanding about their versatile enzyme activities. The directionality of substrate binding is a major mechanistic determinant on the obtained reaction product when comparing TTMs with AC-IVs (3). As already pointed out, our SSN analysis with a slightly less stringent alignment scores in the SSN merged the AC-IV-specific cluster with the *Sa*TTM cluster. Also, *Sa*TTM shared ∼29% identity with both TTMs and AC-IVs, so it is difficult to annotate these conserved enzymes solely on sequence similarity, as the function-specific features of CYTH enzymes have not been well defined. Our cluster analysis of the CYTH superfamily SSN identifies three key motifs that enable us to predict functions for all family members (Fig. 7, Table S1). According to our analysis, a true AC-IV requires a conserved *HF* motif that extends the N-terminal signature *ExExK* motif to *HFxxxxExExK*, a feature that is absent in all other CYTH enzymes. In the crystal structure of *Yp*CyaB the phenylalanine (F5) is involved in π–π stacking of the adenine base, while the histidine (H4) residue stacks to the phenylalanine *via* cation–π interaction, suggesting a role in binding ATP in the correct orientation. When F5 is mutated in an AC-IV family member the catalytic activity was 8-fold reduced for k_cat_ (7). In addition, the adenine base stacks to the phenylalanine of the *HF* motif within a relatively hydrophobic local environment, but no clear directional contacts like hydrogen bonds are made to the adenine amino group indicating that these AC-IVs act not strictly as adenylyl cyclases. The key *HF* motif is absent in the sequence of all other CYTH proteins; hence, we infer that, in the absence of an N-terminal *HF* motif, the CYTH enzyme function is either TTM or ThTPase.

On the other hand, the *W/YxRxR* motif defines the ThTPases, as this subfamily has a conserved aromatic residue (W53 in case of mouse ThTPase) that is capable of stacking the aromatic thiazole moeity of thiamine. Mutation of this residue reduced the plasticity of the ThTPase (6). We find this motif only in one other subfamily (cluster 7) that contains a bi-domain architecture harboring enzymes of the plant TTM1/2 type, which are “TTMs” with a strong substrate preference for pyrophosphate (18). All other clusters have small amino acid substitution at this position (A54 for *Sa*TTM and S59 for *Yp*CyaB). Two arginine residues (R55 and R57 for mouse ThTPase) are required for interaction with the β- and γ-phosphates of ThTP. In AC-IVs, the first arginine residue in this motif is substituted by a small amino acid (V61 in case of *Yp*CyaB), mostly cysteine, valine, or leucine (Fig. S8). Hence, it can be hypothesized that the conserved arginine, which interacts with the γ-phosphate in TTM/ThTPases, is a gate keeper against NTP binding in the cyclase orientation, as the guanidinium group otherwise sterically clashes with the ribose moiety (Fig. 7). The third characteristic feature is an *ExE* motif on strand β8 close to the C terminus of CYTH proteins (E135 and E137 for *Sa*TTM). These conserved glutamate (E) residues are required for metal binding. In TTM/ThTPases, the second glutamate residue is required for metal 2 binding, which is strictly substituted to alanine in case of AC-IVs. Although this contradicts the generic binding of a second metal ion, a two-metal AC-IV mechanism was suggested before for this subfamily (7). Taken together, this combined analysis provides a basis to delineate proper functional annotation of all CYTH domain-containing orthologs.

### Biological role of CYTH

The CYTH superfamily proteins exist in all kingdom of life and can be traced back to the last common ancestor, but the physiological function of this protein family has largely remained elusive. It has been proposed that members of the CYTH superfamily might be involved in the interface of metabolism of phosphoryl compounds and nucleotides. Deletion mutants of the founding member of the CYTH protein superfamily showed no significant cellular defects (21).

So far, the best characterized CYTH proteins from a functional viewpoint derive from plants. The *Arabidopsis* TTM3 (*At*TTM3) knockout plant showed an anomalous root phenotype, indicating a potential role in root development (22). Although it has been established that plant TTMs act as a polyphosphate hydrolase, recent reports suggest that *Brachypodium distachyon* and *Hippeastrum x hybridum* TTM proteins might be able to produce a low amount of cAMP under laboratory conditions (23, 24). In our enzyme function–based cluster analysis (Fig. 1) we identified that all of the so-far characterized plant TTMs were present in the same cluster (cluster 9). As stated above, *At*TTM1/2 from cluster 7 are different from the classical TTMs, as they lack the signature motifs for triphosphate binding and γ-phosphate hydrolysis (*ExExK* and *ExE*, see Table S1). This cluster still harbors the ThTPase typical *W/YxRxR* motif that is involved in substrate binding from the metal-opposing site, as well as the described *DxY* motif required for α/β-water placement (Figs. 6 and S8). This goes along with the observation that *At*TTM2 exhibits pyrophosphatase activity *in vitro*. Upon their deletion, plants became hypersensitive to both virulent and avirulent pathogens and accumulate elevated levels of sialic acid upon infection (25). On the other hand, *At*TTM1 that holds a similar enzyme activity as *At*TTM2 was shown to be rather involved in leaf senescence (18). These contrasting findings are food for future studies on the regulatory and metabolic roles of plant TTMs.

The strictly cAMP-producing CYTH enzymes, the AC-IVs, formed only a small cluster in the whole CYTH SSN (Fig. 1, cluster 10). The biological role of AC-IV is yet to be established. The seminal work discovering AC-IV in *Aeromonas hydrophila* showed that the *cyaB* gene is not expressed under standard growth conditions. However, it can complement the function of another adenylyl cyclase, *cyaA*, when overproduced in a *cyaA* deletion strain. The *cyaB* mutant strain had no visible defects and behaved similar to the wildtype strain. Cellular cAMP levels were also noted to be unaltered upon deletion under normal growth condition (21). In our SSN analysis we identified that the huge majority of organisms that harbor an AC-IV are (potential) pathogens. Hence it is conceivable to envision that these AC-IVs might be repressed during normal growth and play no function on cellular cAMP production, but participate during pathogenesis. AC-IV enzymes possess no similarities with other bacterial toxins and are devoid of any signal sequence (21). Clearly, future studies will be required to address how expression of AC-IV enzymes is repressed under normal growth condition and what their precise role during host infection and pathogen maintenance.

In this study we showed that the CYTH enzyme in crenarchaeote *S. acidocaldarius* previously annotated as adenylyl cyclase is actually a phosphohydrolase of the TTM family. *Sa*TTM prefers inorganic triphosphate substrate over NTPs. Inorganic polyphosphates are considered as energy source readily available for converting di- or monophosphorylated nucleotides to more accessible triphosphorylated ones (like ATP), as metal chelating polymers or as buffers for cellular pH homeostasis (26). In bacteria, polyphosphates are formed by PolyP kinase, whose deletion mutants are defective in biofilm formation, quorum sensing, motility, and other virulent properties (27). Electron dense amorphous PolyP granules were noted in the archaeal counterpart (28, 29, 30). PolyP accumulation in archaea is directly linked to metal toxicity (31) and oxidative stress (32), and its production is dependent on inorganic phosphate availability in *Methanosarcina mazei* (33). The exopolyphosphatase (PPX) gene was identified in *Sulfolobales*, whereas the polyP kinase is yet to be identified (34). In a recent study the overproduced *ppx* gene in *S. acidocaldarius* led to a mutant strain lacking PolyP, which was impaired in biofilm formation, motility, archaellum assembly, and adhesion to glass surfaces (35). Among extremophiles polyP might play a crucial role in environmental fitness (34). Although the concentration of triphosphates is not known in *S. acidocaldarius*, but considering our present finding and previous reports, we envision that TTM in archaea might be directly involved in the polyP metabolism and will indirectly take part in PolyP-mediated regulatory processes and oxidative stress response.

## Experimental procedures

### Protein overproduction and purification

*Sa*TTM (*Saci_*0718) ORF amplified from *S. acidocaldarius* DSM 639 genome having *Nco*I and *Bam*HI restriction enzyme sites was cloned into *Nco*I/*Bam*HI-digested T7-RNA polymerase–based expression plasmid pSA4 (36) to generate pSVA1028 that upon induction with IPTG produces C-terminally His_6_-fusion of *Sa*TTM. Site-directed mutants were produced using “around-the-horn” PCR (37) of the pSVA1028. The correct DNA sequences were verified by sequencing.

*E. coli* BL21-Gold (DE3) was used for heterologous overproduction of C-terminal His_6_-fused *Sa*TTM. Cells were grown in lysogeny broth supplemented with 100 μg/ml ampicillin at 37 °C until an *A*_600_ of 0.5. The overproduction of *Sa*TTM was induced by using 0.75 mM IPTG at 18 °C overnight. Cells from 2 L culture were harvested (3200*g* for 20 min), resuspended in 30 ml lysis buffer (200 mM NaCl, 20 mM Hepes, pH 8.0), and subsequently lysed using French Press. The lysate was incubated at 70 °C for 10 min to reduce *E. coli* contaminants, and the heat-stable cell-free lysate was collected using 39,000*g* for 20 min. The supernatant was loaded on a 5-ml Ni-NTA column (Protino) and washed with six column volumes of lysis buffer before eluting the protein with four column volumes of elution buffer (lysis buffer with 500 mM imidazole). The elution was concentrated and further purified by size exclusion chromatography using a HiLoad 26/600 Superdex 200 PG (GE Healthcare) column equilibrated with SEC buffer (200 mM NaCl, 50 mM Tris, pH 9.0). *Sa*TTM protein purity was monitored using a Coomassie-stained 15% SDS-PAGE. The oligomerization of *Sa*TTM was checked on an analytical Superdex 200 10/300 GL column and using the SEC buffer system. Pure protein was concentrated to 2.5 to 5 mg/ml using ultrafiltration and stored at −80 °C until activity assays or crystallization.

### Crystallization of *Sa*TTM

To obtain *Sa*TTM crystals 10 mg/ml protein in SEC buffer was mixed with the respective precipitant in an equal volume ratio. Crystals were grown at 4 °C using sitting-drop vapor diffusion. For the ATP state, 10 mM CaCl_2_ and 5 mM ATP were added to the protein solution. The best diffracting crystal appeared in condition JCSG Core IE7 (0.2 M calcium acetate, 0.1 M MES pH 6.0, 20% (w/v) PEG 8000). The open state of *Sa*TTM (PDB: 7NS8) originated from the ATP screen in a precipitant condition with 0.2 M lithium sulfate, 0.1 M CAPS pH 10.5, 2 M ammonium sulfate. High concentration of sulfate in the precipitation condition competed out the phosphate substrate from the active site. The Michaelis complex was obtained from a *Sa*TTM mixed with 10 mM CaCl_2_ (or 10 mM MgCl_2_), 0.5 mM MgCl_2_, and 5 mM of hexa-polyphosphate, P_6_i (or tri-polyphosphate, PPPi). Both, PPPi+Ca^2+^ and PPPi+Mg^2+^, crystallized in 0.2 M NaCl, 0.1 M Na/KPO_4_ pH 6.2, 20% (w/v) PEG 1000. Note, the hexa-polyphosphate was highly impure as detected by malachite green assay (data not shown). The product (PPi) inhibited *Sa*TTM in the presence of 10 mM CaCl_2_, and 5 mM PPi was crystallized in 0.17 M ammonium acetate, 0.085 M sodium acetate pH 4.6, 25.5% (w/v) PEG 4000, 15% (v/v) glycerol, and *Sa*TTM in the PPi-divalent metal-free state in the presence of 10 mM MgCl_2_ and 5 mM PPi in 0.2 M potassium acetate, 20% (w/v) PEG 3350. Crystals were harvested at 4 °C as temperature seems to have significant effect on crystallization of *Sa*TTM complexes. Crystals were cryoprotected in mother liquor supplemented with 30% glycerol except the PPPi–Mg^2+^ complex and subsequently flash frozen using liquid nitrogen for data collection.

### Structure solution

X-ray data for different states of *Sa*TTM were collected from single crystals at the ESRF. Crystallography data were processed using iMOSFLM in the CCP4i2 package and XDS (38, 39). Initial phases for *Sa*TTM⋅ATP⋅Ca^2+^ were obtained using a *SWISS model* (40) of *Sa*TTM, based on PDB entry 2EEN (identity 39%) and its closed-state domain as a search model for molecular replacement in Phaser-MR (41). Atomic models of the other crystallized states of *Sa*TTM were obtained by using the structure of *Sa*TTM in complex with ATP and calcium as a search model for Phaser-MR. Iterative model building and refinements were performed with COOT and phenix.refine (42, 43). Data collection and refinement statistics are summarized in Table S2. The resulting coordinates were deposited at the Protein Data Bank under accession numbers 7NS8, 7NS9, 7NSA, 7NSF, 7NSD, and 7OA2. The structural analysis and figure generation was performed with UCSF Chimera (44).

### Activity assay

Triphosphatase activity of *Sa*TTM was analyzed with the Malachite Green Phosphate Assay Kit (MAK307-1KT) from Sigma-Aldrich according to manufacturer's protocol. Reactions were quenched by mixing 80 μl assay mix into 20 μl working reagent using 96-well PS F-bottom microplates (Greiner). After color development for 30 min at room temperature, the absorbance was measured at 620 nm using a plate reader (spectrometer Infinite 200, Tecan). The results were analyzed with Microsoft Excel and GraphPad Prism. The pH optimum of *Sa*TTM was determined by measuring the amount of reaction product (Pi release) from 5 nM *Sa*TTM, 2 mM MgCl_2_, and 0.1 mM PPPi buffered in 50 mM Tris and 200 mM NaCl at varying pH values (6.1–9.1) after 5 min incubation. To determine the preferred metal cofactor, 5 nM *Sa*TTM, 2 mM metal ion (Mg^2+^, Ca^2+^, Mn^2+^, Zn^2+^, Ni^2+^) and 0.1 mM PPPi were incubated at 75 °C for 5 min buffered in 50 mM Tris and 200 mM NaCl at pH 8.6. The preferred substrate was analyzed by using the same parameters for 10 min with Mg^2+^ as a cofactor and varying substrates (PPPi, ATP, GTP, AP_4_A, AP_5_A) at 0.1 mM concentration. Mutant activities were determined accordingly with 5 min incubation. In order to test the product inhibition, as well as the ability of Ca^2+^ to inhibit *Sa*TTM, a titration series of 1 nM to 10 mM inhibitor (PPi or Ca^2+^) was performed by measuring their influence on the product formation of 5 nM *Sa*TTM, 2 mM MgCl_2_, and 0.1 mM PPPi buffered in 50 mM Tris and 200 mM NaCl at pH 8.6 incubated for 5 and 10 min at 75 °C.

To analyze the enzyme kinetics of *Sa*TTM phosphatase activity, 5 nM enzyme was incubated together with 2 mM MgCl_2_ and varying PPPi concentrations (0.005–0.5 mM) incubated in 50 mM Tris and 200 mM NaCl at pH 8.6 and 75 °C. To obtain kinetic parameters, the velocities at different substrate concentrations were determined by measuring the Pi formation at different time points and the resulting linear regressions of four measurements were used for the Michaelis–Menten analysis.

To check products on HPLC, the reactions were diluted 5-fold with double-distilled water and the enzyme was denatured by adding 100 μl chloroform followed by 15 s of vigorous shaking, 15 s heat denaturation at 95 °C and snap freezing in liquid nitrogen. The samples were thawed, the two phases were separated by centrifugation (17,300*g*, 10 min, 4 °C), and 10 μl of the aqueous phase containing the nucleotides was subjected to HPLC analysis. Nucleotides were separated with an anion exchange column (Metrosep A Supp 5 – 150/4.0) with 100 mM (NH_4_)_2_CO_3_ pH 9.25 at a flow rate of 0.7 ml/min as eluent and detected at 260 nm wavelength in agreement with standards.

For the activity analysis of *Sa*TTM toward tetraphosphate, 21 μM *Sa*TTM was incubated in a total volume of 150 μl together with 15 mM Tris pH 7.5 and 4 mM MgCl_2_ for 3 min at 75 °C. The reaction was initiated by the addition of 0.5 mM tetraphosphate and terminated after 10 min through incubation on ice for 10 min, followed by centrifugation in a spin filter to remove the enzyme (4 °C, 35 min, 10,000*g*, Amicon-Ultra 0.5, 10 kDa). The flow through was analyzed by ionic exchange chromatography using a Thermo Scientific Dionex ICS-5000+ equipped with a conductivity detector and an EGC500 eluent generator. A volume of 10 μl of the flow through was injected followed by elution with a flow rate of 0.25 ml/min and a KOH gradient starting from 5 mM KOH. The eluent was raised to 12 mM within 5 min, followed by an increase to 20 mM until 10 min and 70 mM within 15 min. KOH concentration was maintained at 70 mM until 26 min, subsequently lowered to 5 mM at 26.1 min followed by equilibration until termination of the run at 27 min.

### Sequence similarity network

The protein family PF01928 (CYTH) was used as input for the Enzyme Similarity Tool provided by the Enzyme Function Initiative (14) to generate the SSN. Using the domain boundaries and an E-value of 10^−5^, the initial network was generated and subsequently modified by applying higher stringent cut offs (10^−15^, 10^−20^) as alignment scores for the final SSNs. Each node represents proteins with >40% identity. The resulting networks were subjected to the recently implemented cluster analysis for automatic cluster-WEBLOGO generation, and the final networks were analyzed with Cytoscape 3.5.1 (45, 46).

## Data availability

The atomic coordinates of the crystal structures of *Sa*TTM, *Sa*TTM⋅PPPi⋅Ca^2+^, *Sa*TTM⋅PPi⋅Ca^2+^, *Sa*TTM⋅PPPi⋅Mg^2+^, *Sa*TTM⋅ATP⋅Ca^2+^, and *Sa*TTM⋅PPi⋅K^+^ have been deposited in the Protein Data Bank (PDB ID codes 7NS8, 7NS9, 7NSA, 7NSF, 7NSD, and 7OA2, respectively).

## Supporting information

This article contains supporting information.

## Conflict of interest

The authors declare that they have no conflicts of interest with the contents of this article.

## References

[1] Iyer L.M., Aravind L. (2002). The catalytic domains of thiamine triphosphatase and CyaB-like adenylyl cyclase define a novel superfamily of domains that bind organic phosphates. BMC Genomics.

[2] Keppetipola N., Jain R., Shuman S. (2007). Novel triphosphate phosphohydrolase activity of Clostridium thermocellum TTM, a member of the triphosphate tunnel metalloenzyme superfamily. J. Biol. Chem..

[3] Martinez J., Truffault V., Hothorn X.M. (2015). Structural determinants for substrate binding and catalysis in triphosphate tunnel metalloenzymes. J. Biol. Chem..

[4] Lima C.D., Wang L.K., Shuman S. (1999). Structure and mechanism of yeast RNA triphosphatase: An essential component of the mRNA capping apparatus. Cell.

[5] Gong C., Smith P., Shuman S. (2006). Structure-function analysis of Plasmodium RNA triphosphatase and description of a triphosphate tunnel metalloenzyme superfamily that includes Cet1-like RNA triphosphatases and CYTH proteins. Rna.

[6] Delvaux D., Kerff F., Murty M.R.V.S., Lakaye B., Czerniecki J., Kohn G., Wins P., Herman R., Gabelica V., Heuze F., Tordoir X., Marée R., Matagne A., Charlier P., De Pauw E. (2013). Structural determinants of specificity and catalytic mechanism in mammalian 25-kDa thiamine triphosphatase. Biochim. Biophys. Acta.

[7] Gallagher D.T., Kim S.K., Robinson H., Reddy P.T. (2011). Active-site structure of class IV adenylyl cyclase and transphyletic mechanism. J. Mol. Biol..

[8] Steegborn C. (2014). Structure, mechanism, and regulation of soluble adenylyl cyclases - similarities and differences to transmembrane adenylyl cyclases. Biochim. Biophys. Acta Mol. Basis Dis..

[9] Linder J.U. (2006). Class III adenylyl cyclases: Molecular mechanisms of catalysis and regulation. Cell. Mol. Life Sci..

[10] Khannpnavar B., Mehta V., Qi C., Korkhov V. (2020). Structure and function of adenylyl cyclases, key enzymes in cellular signaling. Curr. Opin. Struct. Biol..

[11] Bettendorff L., Wins P. (2013). Thiamine triphosphatase and the CYTH superfamily of proteins. FEBS J..

[12] Baumann A., Lange C., Soppa J. (2007). Transcriptome changes and cAMP oscillations in an archaeal cell cycle. BMC Cell Biol..

[13] Leichtling B.H., Rickenberg H.V., Seely R.J., Fahrney D.E., Pace N.R. (1986). The occurrence of cyclic AMP in archaebacteria. Biochem. Biophys. Res. Commun..

[14] Gerlt J.A., Bouvier J.T., Davidson D.B., Imker H.J., Sadkhin B., Slater D.R., Whalen K.L. (2015). Enzyme function initiative-enzyme similarity tool (EFI-EST): A web tool for generating protein sequence similarity networks. Biochim. Biophys. Acta.

[15] Gallagher D.T., Smith N.N., Kim S.K., Heroux A., Robinson H., Reddy P.T. (2006). Structure of the class IV adenylyl cyclase reveals a novel fold. J. Mol. Biol..

[16] Delvaux D., Murty M.R.V.S., Gabelica V., Lakaye B., Lunin V.V., Skarina T., Onopriyenko O., Kohn G., Wins P., De Pauw E., Bettendorff L. (2011). A specific inorganic triphosphatase from Nitrosomonas europaea: Structure and catalytic mechanism. J. Biol. Chem..

[17] Jain R., Shuman S. (2008). Polyphosphatase activity of CthTTM, a bacterial triphosphate tunnel metalloenzyme. J. Biol. Chem..

[18] Ung H., Karia P., Ebine K., Ueda T., Yoshioka K., Moeder W. (2017). Triphosphate tunnel metalloenzyme function in senescence highlights a biological diversification of this protein superfamily. Plant Physiol..

[19] Zhang Y., Agrebi R., Bellows L.E., Collet J.F., Kaever V., Gründling A. (2017). Evolutionary adaptation of the essential tRNA methyltransferase TrmD to the signaling molecule 3′,5′-cAMP in bacteria. J. Biol. Chem..

[20] Krissinel E., Henrick K. (2007). Inference of macromolecular assemblies from crystalline state. J. Mol. Biol..

[21] Sismeiro O., Trotot P., Biville F., Vivares C., Danchin A. (1998). Aeromonas hydrophila adenylyl cyclase 2: a new class of adenylyl cyclases with thermophilic properties and sequence similarities to proteins from hyperthermophilic archaebacteria. J. Bacteriol..

[22] Moeder W., Garcia-Petit C., Ung H., Fucile G., Samuel M.A., Christendat D., Yoshioka K. (2013). Crystal structure and biochemical analyses reveal that the Arabidopsis triphosphate tunnel metalloenzyme AtTTM3 is a tripolyphosphatase involved in root development. Plant J..

[23] Świeżawska B., Duszyn M., Kwiatkowski M., Jaworski K., Pawełek A., Szmidt-Jaworska A. (2020). Brachypodium distachyon triphosphate tunnel metalloenzyme 3 is both a triphosphatase and an adenylyl cyclase upregulated by mechanical wounding. FEBS Lett..

[24] Świezawska B., Jaworski K., Pawełek A., Grzegorzewska W., Szewczuk P., Szmidt-Jaworska A. (2014). Molecular cloning and characterization of a novel adenylyl cyclase gene, HpAC1, involved in stress signaling in Hippeastrum x hybridum. Plant Physiol. Biochem..

[25] Ung H., Moeder W., Yoshioka K. (2014). Arabidopsis triphosphate tunnel metalloenzyme2 is a negative regulator of the salicylic acid-mediated feedback amplification loop for defense responses. Plant Physiol..

[26] Brown M.R.W., Kornberg A. (2004). Inorganic polyphosphate in the origin and survival of species. Proc. Natl. Acad. Sci. U. S. A..

[27] Rashid M.H., Kornberg A. (2000). Inorganic polyphosphate is needed for swimming, swarming, and twitching motilities of *Pseudomonas aeruginosa*. Proc. Natl. Acad. Sci. U. S. A..

[28] Toso D.B., Henstra A.M., Gunsalus R.P., Zhou Z.H. (2011). Structural, mass and elemental analyses of storage granules in methanogenic archaeal cells. Environ. Microbiol..

[29] Toso D.B., Javed M.M., Czornyj E., Gunsalus R.P., Zhou Z.H. (2016). Discovery and characterization of iron sulfide and polyphosphate bodies coexisting in *Archaeoglobus fulgidus* Cells. Archaea.

[30] Scherer P.A., Bochem H.P. (1983). Ultrastructural investigation of 12 Methanosarcinae and related species grown on methanol for occurrence of polyphosphatelike inclusions. Can. J. Microbiol..

[31] Remonsellez F., Orell A., Jerez C.A. (2006). Copper tolerance of the thermoacidophilic archaeon *Sulfolobus metallicus*: Possible role of polyphosphate metabolism. Microbiology.

[32] Li L., Li Q., Rohlin L., Kim U.M., Salmon K., Rejtar T., Gunsalus R.P., Karger B.L., Ferry J.G. (2007). Quantitative proteomic and microarray analysis of the archaeon *Methanosarcina acetivorans* grown with acetate versus methanol. J. Proteome Res..

[33] Paula F.S., Chin J.P., Schnürer A., Müller B., Manesiotis P., Waters N., Macintosh K.A., Quinn J.P., Connolly J., Abram F., McGrath J.W., O’Flaherty V. (2019). The potential for polyphosphate metabolism in Archaea and anaerobic polyphosphate formation in *Methanosarcina mazei*. Sci. Rep..

[34] Orell A., Navarro C.A., Rivero M., Aguilar J.S., Jerez C.A. (2012). Inorganic polyphosphates in extremophiles and their possible functions. Extremophiles.

[35] Recalde A., van Wolferen M., Sivabalasarma S., Albers S.-V., Navarro C.A., Jerez C.A. (2021). The role of polyphosphate in motility, adhesion, and biofilm formation in Sulfolobales. Microorganisms.

[36] Albers S.V., Szabó Z., Driessen A.J.M. (2003). Archaeal homolog of bacterial type IV prepilin signal peptidases with broad substrate specificity. J. Bacteriol..

[37] Liu H., Naismith J.H. (2008). An efficient one-step site-directed deletion, insertion, single and multiple-site plasmid mutagenesis protocol. BMC Biotechnol..

[38] Potterton L., Agirre J., Ballard C., Cowtan K., Dodson E., Evans P.R., Jenkins H.T., Keegan R., Krissinel E., Stevenson K., Lebedev A., McNicholas S.J., Nicholls R.A., Noble M., Pannu N.S. (2018). CCP 4 i 2: The new graphical user interface to the CCP 4 program suite. Acta Crystallogr. D Struct. Biol..

[39] Kabsch W. (2010). Xds. Acta Crystallogr. D Biol. Crystallogr..

[40] Waterhouse A., Bertoni M., Bienert S., Studer G., Tauriello G., Gumienny R., Heer F.T., De Beer T.A.P., Rempfer C., Bordoli L., Lepore R., Schwede T. (2018). SWISS-MODEL: Homology modelling of protein structures and complexes. Nucleic Acids Res..

[41] McCoy A.J., Grosse-Kunstleve R.W., Adams P.D., Winn M.D., Storoni L.C., Read R.J. (2007). Phaser crystallographic software. J. Appl. Crystallogr..

[42] Afonine P.V., Grosse-Kunstleve R.W., Echols N., Headd J.J., Moriarty N.W., Mustyakimov M., Terwilliger T.C., Urzhumtsev A., Zwart P.H., Adams P.D. (2012). Towards automated crystallographic structure refinement with phenix.refine. Acta Crystallogr. D Biol. Crystallogr..

[43] Emsley P., Lohkamp B., Scott W.G., Cowtan K. (2010). Features and development of Coot. Acta Crystallogr. D Biol. Crystallogr..

[44] Pettersen E.F., Goddard T.D., Huang C.C., Couch G.S., Greenblatt D.M., Meng E.C., Ferrin T.E. (2004). UCSF Chimera - a visualization system for exploratory research and analysis. J. Comput. Chem..

[45] Cline M.S., Smoot M., Cerami E., Kuchinsky A., Landys N., Workman C., Christmas R., Avila-Campilo I., Creech M., Gross B., Hanspers K., Isserlin R., Kelley R., Killcoyne S., Lotia S. (2007). Integration of biological networks and gene expression data using cytoscape. Nat. Protoc..

[46] Crooks G.E., Hon G., Chandonia J.M., Brenner S.E. (2004). WebLogo: A sequence logo generator. Genome Res..

