# Supplementary material for: The archaeal triphosphate tunnel metalloenzyme SaTTM defines structural determinants for the diverse activities in the CYTH protein family
Source: J Biol Chem. 2021 May 23;297(1):100820. doi: 10.1016/j.jbc.2021.100820 (PMC8233210; doi:10.1016/j.jbc.2021.100820)
Supplement: Figures S1–S8; Tables S1 and S2 [file mmc1.pdf]

Supporting information

"The archaeal triphosphate tunnel metalloenzyme *Sa*TTM defines structural determinants for the diverse activities in the CYTH protein family"

Marian S. Vogt[1], Roi R. Ngouoko[1], Michael K. F. Mohr[2], Sonja-Verena Albers[3], Lars-Oliver Essen[1,4]*, Ankan Banerjee[1,5]*

[1]Department of Chemistry, Philipps-Universität Marburg, Hans-Meerwein-Str. 4, D-35032 Marburg, Germany.

[2]Institute of Pharmaceutical Sciences, Albert-Ludwigs-Universität Freiburg, Albertstr. 25, D-79104 Freiburg, Germany

[3]Institute of Biology II, Molecular Biology of Archaea, Albert-Ludwigs-Universität Freiburg, Schänzlestrasse 1, D-79104 Freiburg, Germany.

[4]Center for Synthetic Microbiology, Philipps-Universität Marburg, Hans-Meerwein-Str. 4 D-35032 Marburg

[5]Department of Genetics, Philipps-Universität Marburg, Karl-Von-Frisch-Str 10, D-35043 Marburg, Germany.

*Corresponding author: Ankan Banerjee (banerjee@staff.uni-marburg.de) and Lars-Oliver Essen (essen@chemie.uni-marburg.de)

Supplementary figures

Figure S1

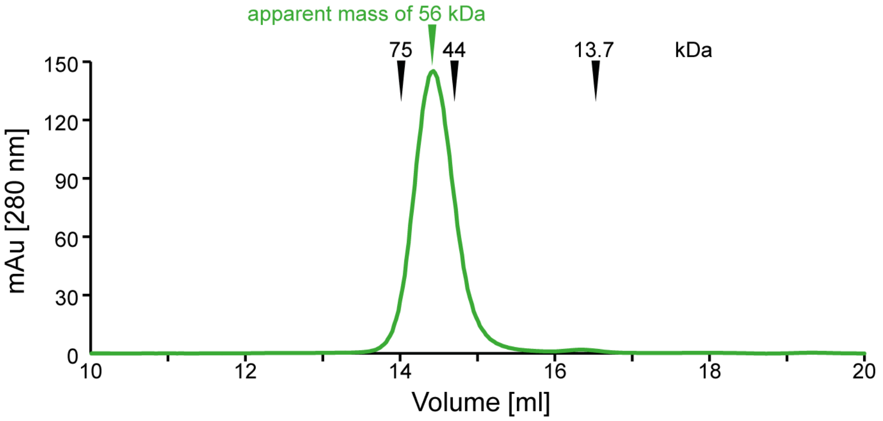

Figure S1 Calibrated SEC run of *Sa*TTM on Biorad SEC650 column. Conalbumin (75 kDa), ovalbumin (44 kDa), and ribonuclease A (13.7 kDa) were used as mass standards. The calculated molecular mass of the *Sa*TTM monomer is 23.65 kDa, its SEC elution corresponds to a dimer in solution.

Figure S2

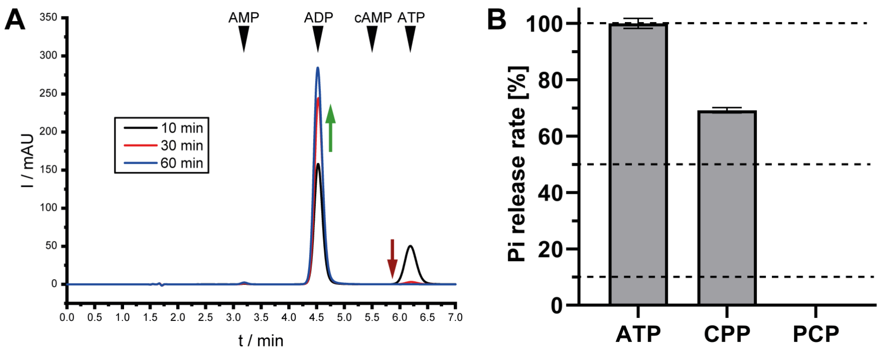

Figure S2 *Sa*TTM converts ATP to ADP by releasing γ-phosphate. A Chromatograms of the time course of *Sa*TTM hydrolyzing ATP to ADP is shown. As indicated by standards, no cAMP is produced. Arrows indicate the decrease (red) of substrate increase (green) of product over time. **B** *Sa*TTM can utilize ATP and the non-hydrolysable ATP analog AMPCPP, but not AMPPCP, as a substrate.

Figure S3

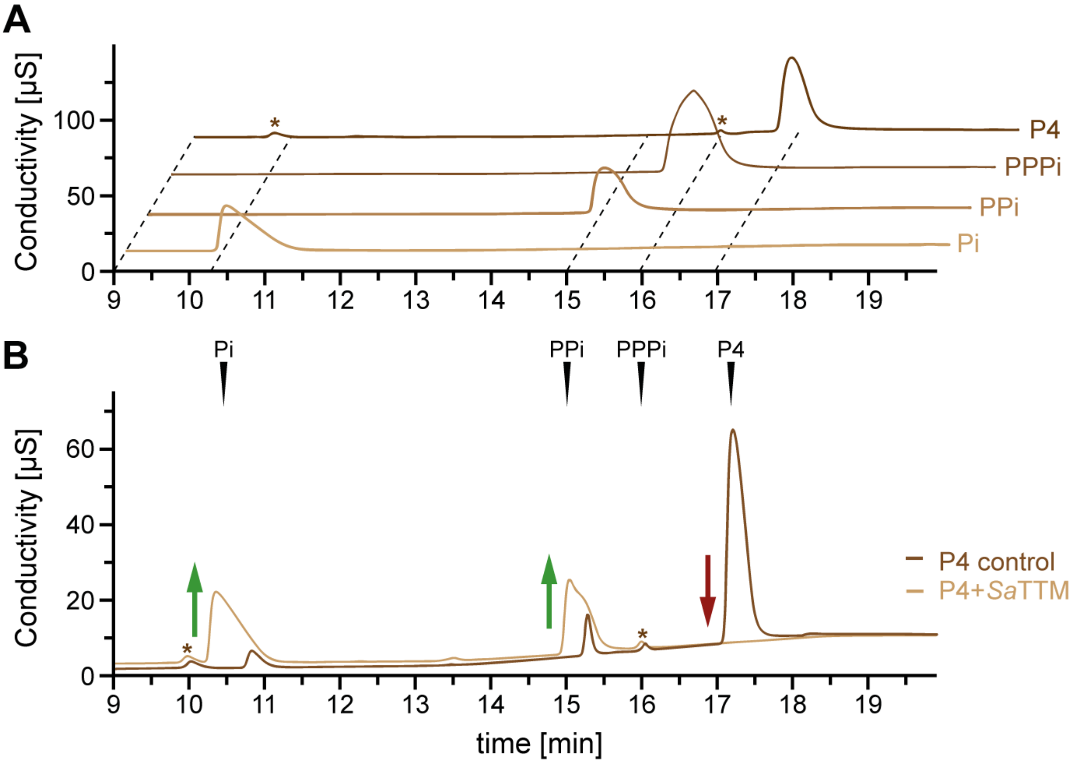

Figure S3 *Sa*TTM can hydrolyze tetraphosphate. A Chromatograms of standards are shown for phosphate (Pi), pyrophosphate (PPi), triphosphate (PPPi), and tetraphosphate (P4). **B** Chromatograms of P4 after incubation alone (brown) and together with *Sa*TTM (ocher) show a complete turnover of P4 as indicated by the red arrow, while the products Pi and PPi increase (green arrows). The two unidentified peaks indicated by asterisks appear throughout the ion exchange chromatography run of P4 without being affected by the sample.

Figure S4

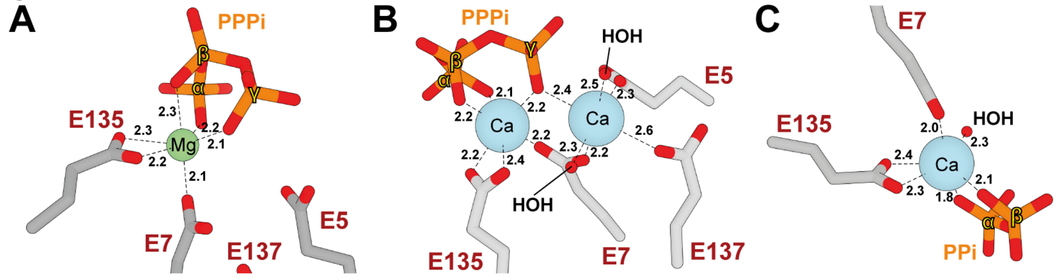

Figure S4 Distorted pseudo-octahedral metal ion coordination in *Sa*TTM. The metal coordination of *Sa*TTM PPPi•Mg^{2+} (**A**), PPPi•Ca^{2+} (**B**), and PPi•Ca^{2+} (**C**) is shown indicated by the dashed lines and the respective distances shown in Angstrom. α-, β-, and γ-positions of phosphates are indicated, so are the residues shown as grey sticks. Oxygens are colored in red, phosphorus in orange, magnesium as a green sphere and calcium as blue sphere. Waters are red spheres as indicated.

Figure S5

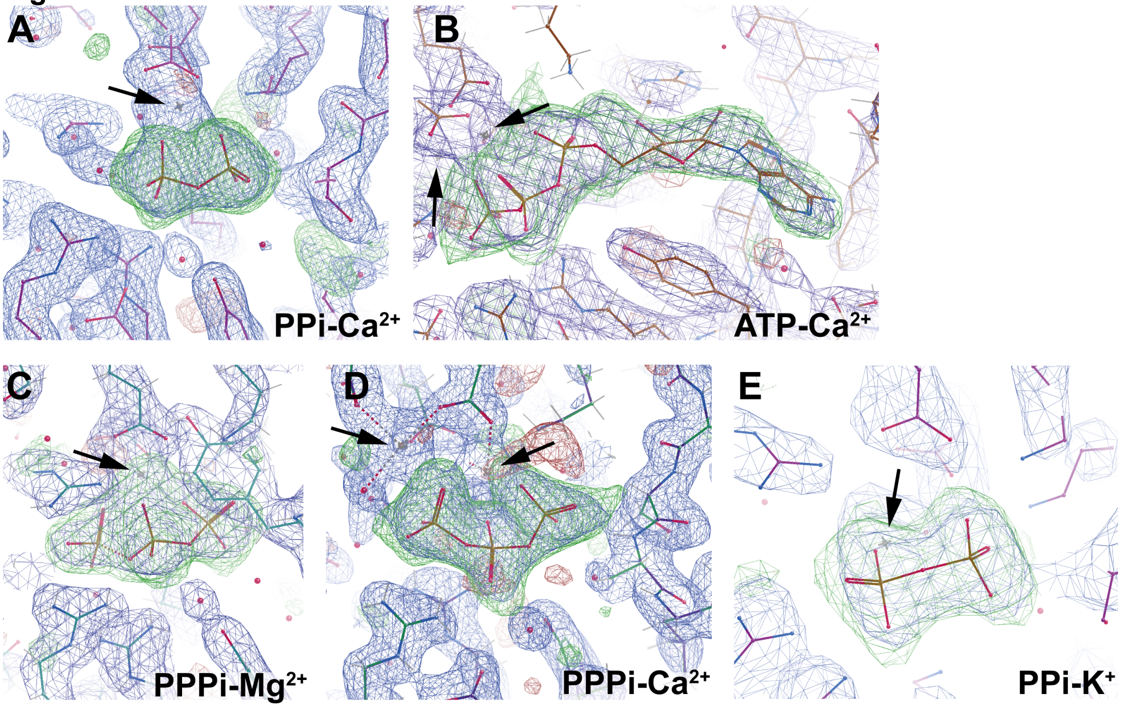

Figure S5 OMIT maps for the different catalytic states of *Sa*TTM. OMIT maps of the respective ligands contoured at σ-level 3.0 are shown in the context of SIGMAA-weighted 2m*F*o-D*F*c maps (blue mesh, contouring level: 1.5) of nearby residues. Arrows highlight the positions of metal ions.

Figure S6

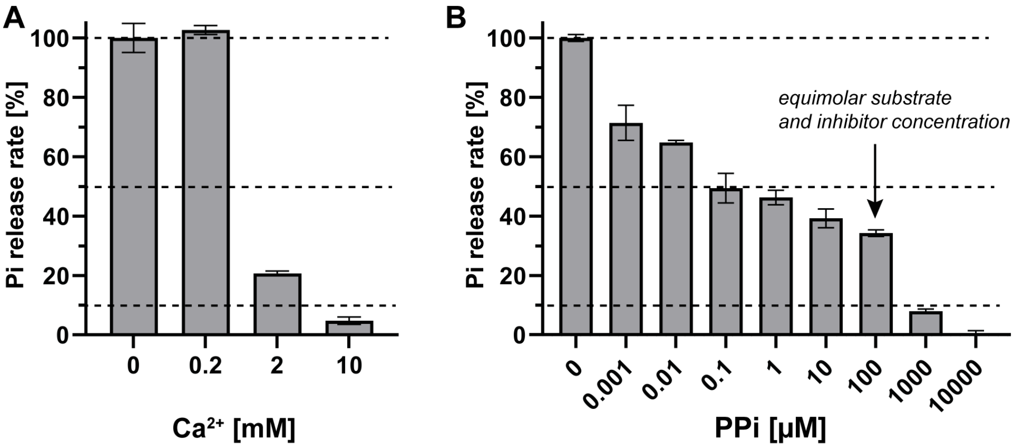

Figure S6 Titrations with inhibitory calcium and pyrophosphate (PPi). A The inhibitory effect of calcium on the triphosphatase activity of *Sa*TTM is shown. **B** The presence of the product PPi has an inhibitory effect on the reaction behavior of *Sa*TTM.

Figure S7

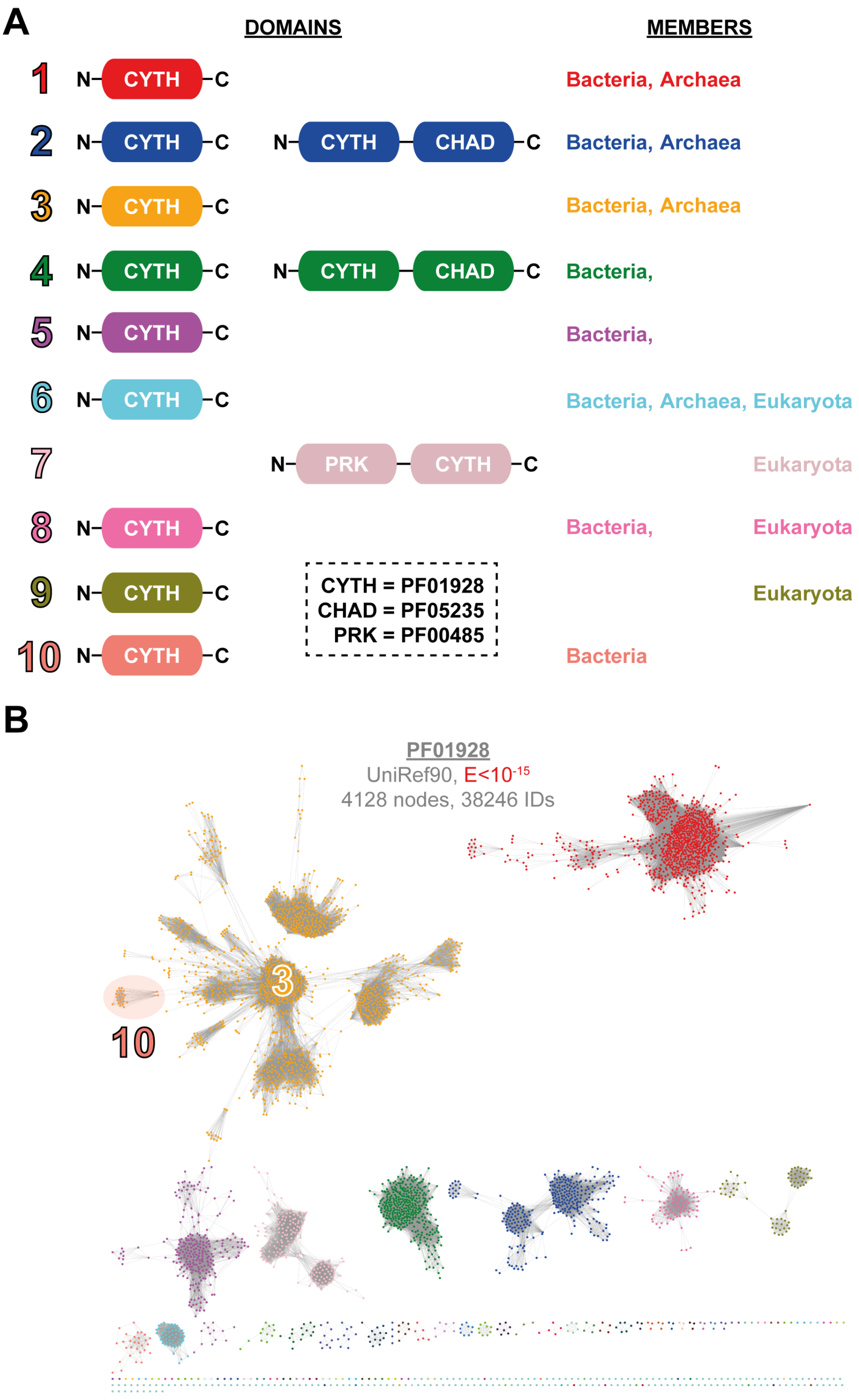

Figure S7 Domain architectures of PF01928 family and its SSN with lower stringency. **A** The prominent domain architectures present in the ten described clusters of the PF01928 family are shown with their appearance among the three kingdoms. The Pfam IDs are noted in the dashed box. **B** The PF01928 SSN with an alignment score of $E<10^{-15}$ is presented. Cluster 10 from the SSN in Fig. 1 is now member of cluster 3 (highlighted in transparent red).

Figure S8 WEBLOGOS of PF01928 clusters 1-10.

WEBLOGO Cluster 1 comprising 868 entries from 2892 IDs within the SSN

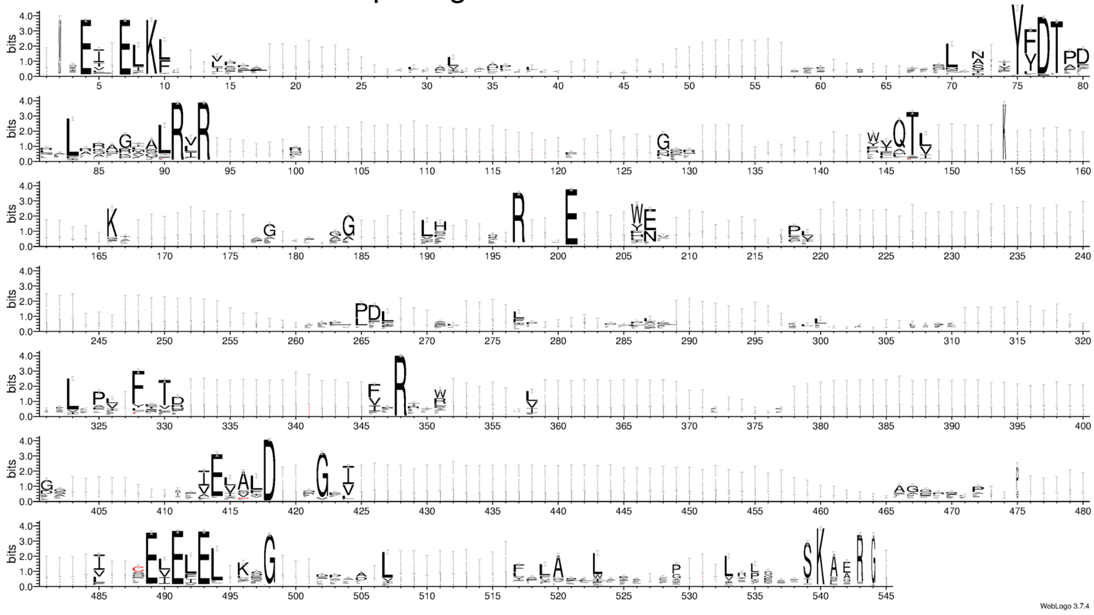

WEBLOGO Cluster 2 comprising 297 entries from 3199 IDs within the SSN

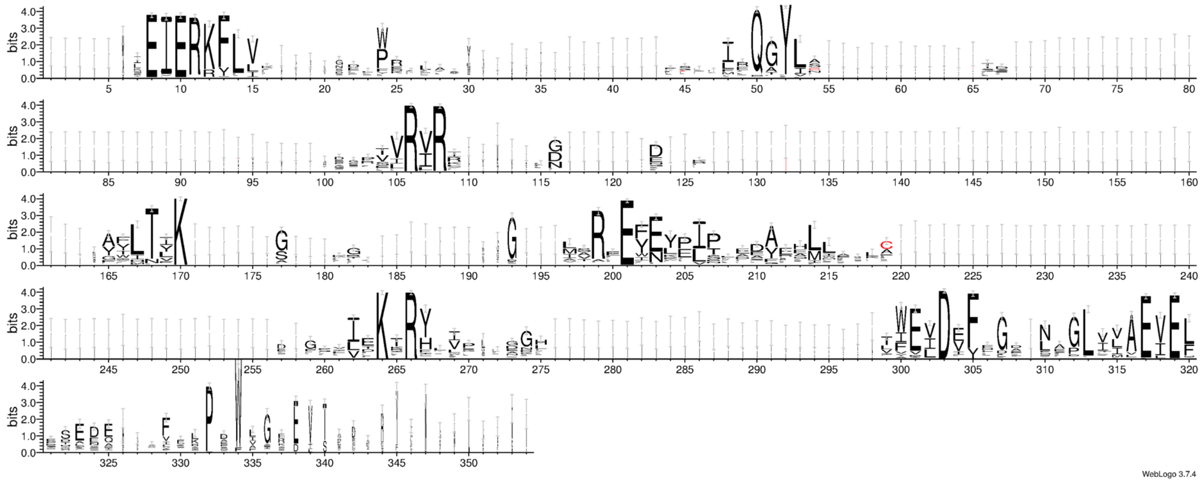

WEBLOGO Cluster 3 comprising 1062 entries from 2430 IDs within the SSN

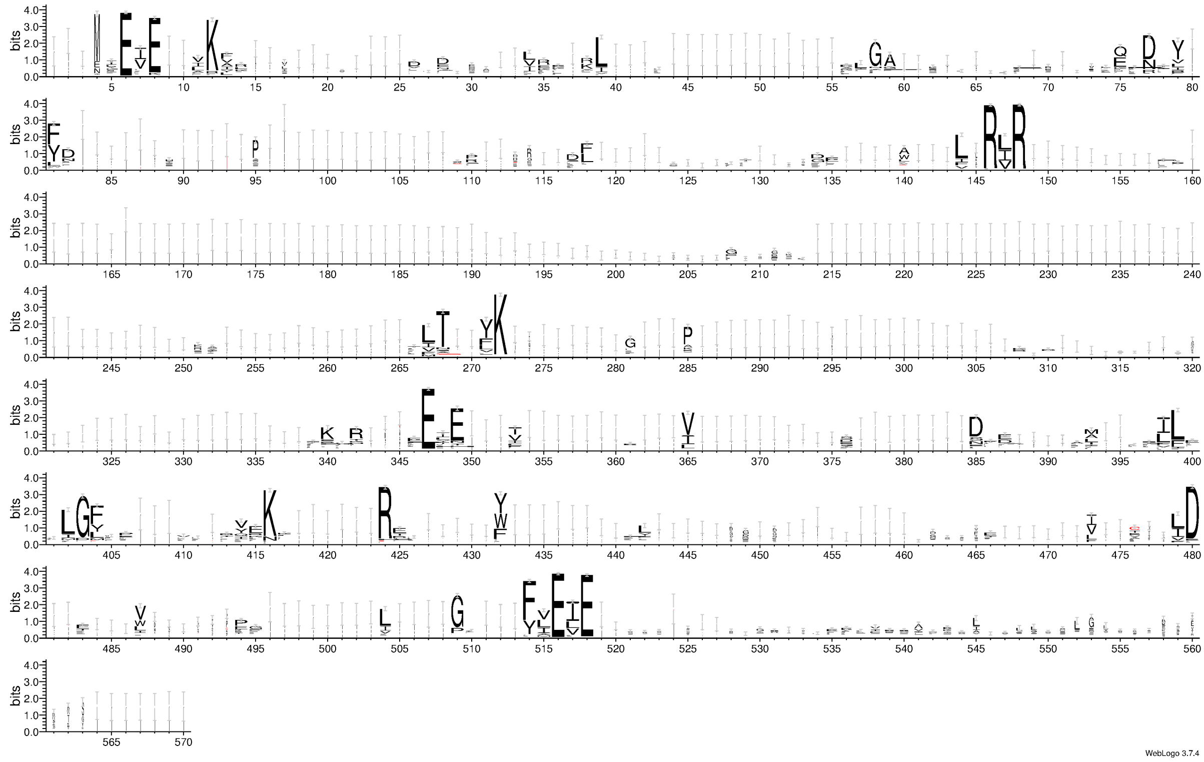

WEBLOGO Cluster 4 comprising 355 entries from 1644 IDs within the SSN

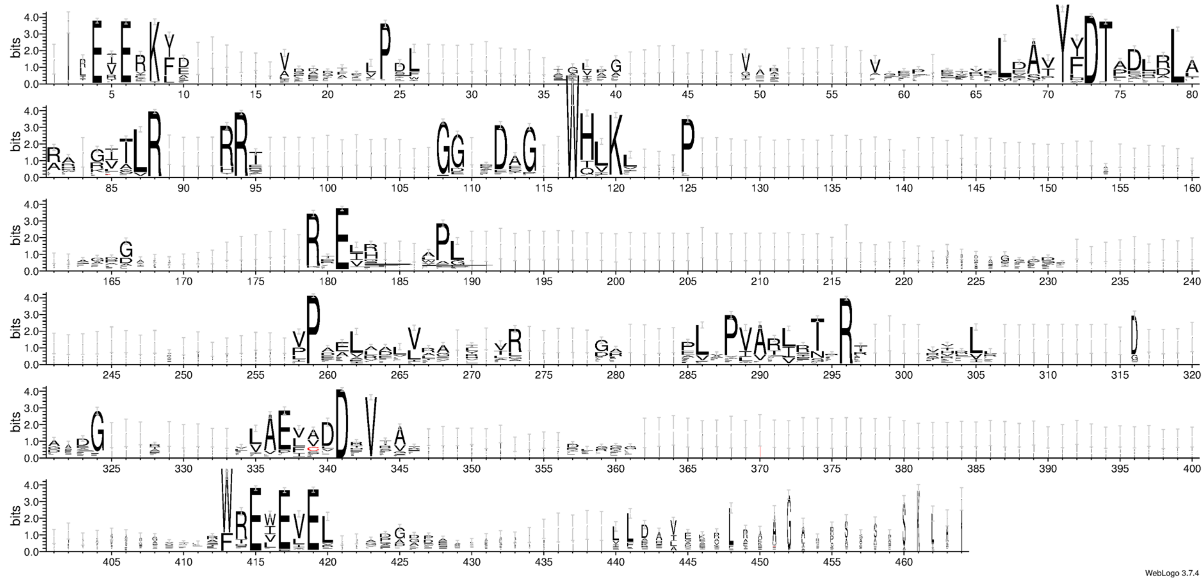

WEBLOGO Cluster 5 comprising 229 entries from 1001 IDs within the SSN

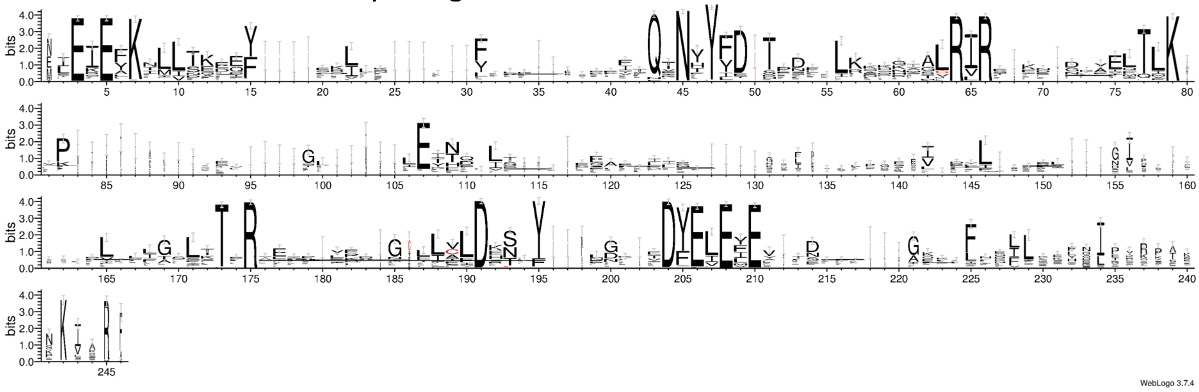

WEBLOGO Cluster 6 comprising 287 entries from 904 IDs within the SSN

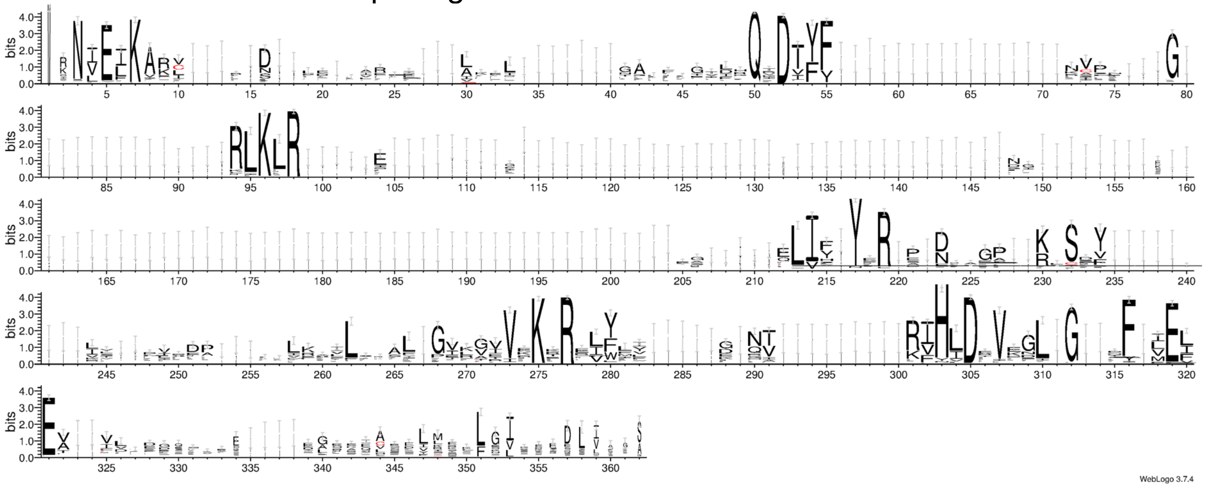

WEBLOGO Cluster 7 comprising 56 entries from 234 IDs within the SSN

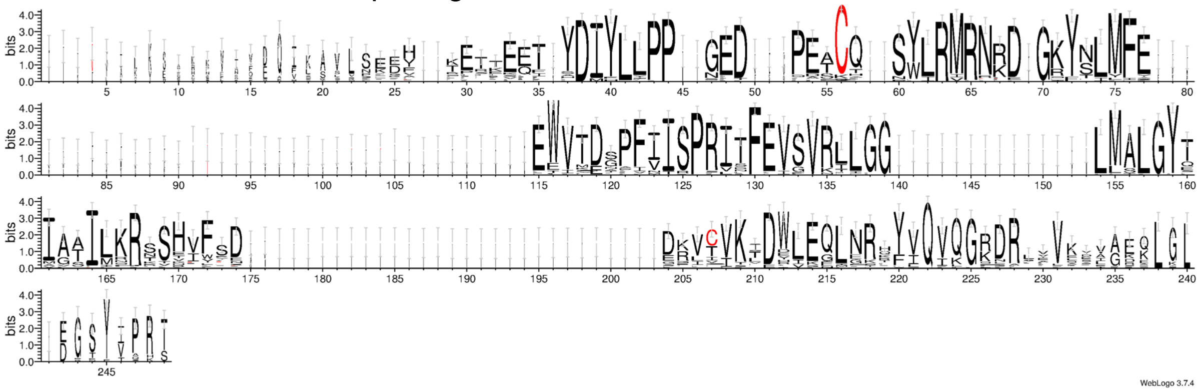

WEBLOGO Cluster 8 comprising 178 entries from 297 IDs within the SSN

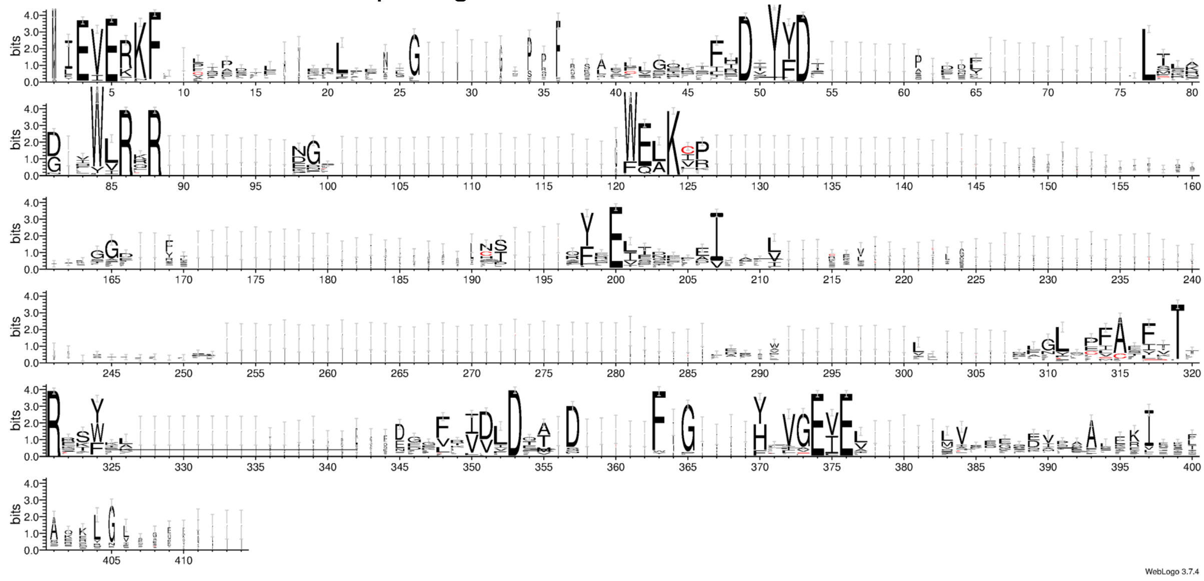

WEBLOGO Cluster 9 comprising 85 entries from 221 IDs within the SSN

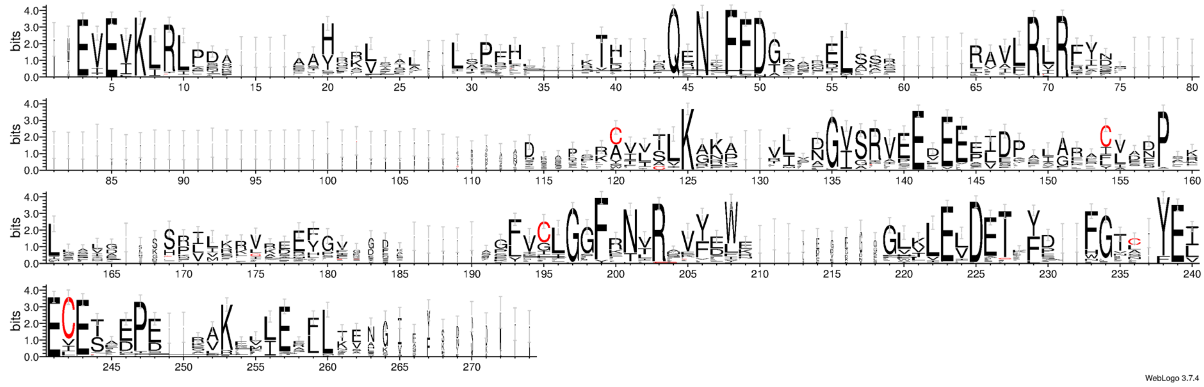

WEBLOGO Cluster 10 comprising 23 entries from 141 IDs within the SSN from the full length alignment, as the HF-motif exceeds the domain boundaries

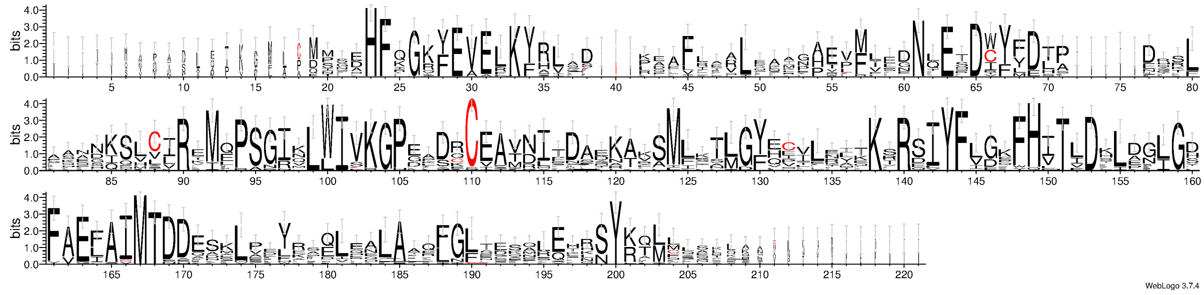

Supplementary tables

Table S1: Overview of characterized PF01928 enzymes. Coloring according to SSN.

PDB	UNIPROT	ORGANISM	NAME	LIGAND/STATE/NOTES	MOTIFS	FUNCTION	SOURCE	CLUSTER
5A60	P30871	*E. coli*	Ygif	PPPi+Mg+Mg	ExExK, GxRxR, ExE	TTM	Martinez et al (2015)	1
5A61	P30871	*E. coli*	Ygif	PPPi+Mn+Mn	ExExK, GxRxR, ExE	TTM	Martinez et al (2015)	1
2FBL, 3TYP	Q82UI9	*N. europaea*	*Neu*TTM	empty, open	ExExK, ExRxR, ExE	TTM	Delvaux et al (2011)	2
	A3DIJ8	*C. thermocellum*	*Cth*TTM		ExExK, SxRxR, ExE	TTM	Jain and Shuman (2008)	2
2DC4	O58740	*P. horikoshii*	*Pho*TTM	3xCl-	ExExK, LxRxR, DxE	TTM	unpublished	3
2EEN	O59483	*P. horikoshii*	*Pho*TTM	empty	ExExK, AxRxR, ExE	TTM	unpublished	3
1YEM	Q8U2H2	*P. furiosus*	*Pfu*TTM	UNK, density for PPPi	ExExK, LxRxR, DxE	TTM	unpublished	3
7NS8	Q4JAT2	*S. acidocaldarius*	*Sa*TTM	2xSO4	ExExK, AxRxR, ExE	TTM	This study	3
7NSF	Q4JAT2	*S. acidocaldarius*	*Sa*TTM	PPPi, Mg	ExExK, AxRxR, ExE	TTM	This study	3
7NS9	Q4JAT2	*S. acidocaldarius*	*Sa*TTM	PPPi, 2x Ca	ExExK, AxRxR, ExE	TTM	This study	3
7NSA	Q4JAT2	*S. acidocaldarius*	*Sa*TTM	PPi, Ca	ExExK, AxRxR, ExE	TTM	This study	3
7NSD	Q4JAT2	*S. acidocaldarius*	*Sa*TTM	ATP, 2x Ca	ExExK, AxRxR, ExE	TTM	This study	3
7OA2	Q4JAT2	*S. acidocaldarius*	*Sa*TTM	PPi, K	ExExK, AxRxR, ExE	TTM	This study	3
3SY3	A0A1J9V1U0	*B. antharcis*		empty	ExExK, AxRxR, ExE	TTM	unpublished	5
3TJ7	A0A1J9V1U0	*B. antharcis*		empty, AMP stacked in interface	ExExK, AxRxR, ExE	TTM	unpublished	5
2GFG	Q9K901	*B. halodurans*		UNL	ExExK, AxRxR, ExE	TTM	unpublished	5
	A0A0H2XGC0	*S. aureus*	SAUSA300_0905		ExExK, AxRxR, ExE	TTM	Zhang et al (2017)	5
	Q9C9B9	*A. thaliana*	*At*TTM1		no ExExK, Y/WxRxR, no ExE	PPase	Ung et al (2017)	7
	Q9C664	*A. thaliana*	*At*TTM2		no ExExK, Y/WxRxR, no ExE	PPase	Ung et al (2017)	7
3BHD	Q9BU02	*H. sapiens*	*Hs*ThTPase	SO4, CIT, Cl	ExExK, WxRxR, ExE	ThTPase	unpublished	8
3TVL	Q9BU02	*H. sapiens*	*Hs*ThTPase	PPPi	ExExK, WxRxR, ExE	ThTPase	Delvaux et al (2013)	8
2JMU	Q8JZL3	*M. musculus*	*Mm*ThTPase	empty (NMR)	ExExK, WxRxR, ExE	ThTPase	Song et al (2008)	8
5A64	Q8JZL3	*M. musculus*	*Mm*ThTPase	Thiaminetriphosphate (water instead of M1 build, small offset)	ExExK, WxRxR, ExE	ThTPase	Martinez et al (2015)	8
5A65	Q8JZL3	*M. musculus*	*Mm*ThTPase	Thiaminepyrophosphate +Pi+2Mg	ExExK, WxRxR, ExE	ThTPase	Martinez et al (2015)	8
3V85	Q9SIY3	*A. thaliana*	*At*TTM3	CIT	ExExK, VxRxR, ExE	TTM	unpublished	9
5A5Y	Q9SIY3	*A. thaliana*	*At*TTM3	PPPi, Mg	ExExK, VxRxR, ExE	TTM	Martinez et al (2015)	9
5A66	Q9SIY3	*A. thaliana*	*At*TTM3	PPPi, Mn	ExExK, VxRxR, ExE	TTM	Martinez et al (2015)	9
5A67	Q9SIY3	*A. thaliana*	*At*TTM3	PPPi, Mn	ExExK, VxRxR, ExE	TTM	Martinez et al (2015)	9
5A68	Q9SIY3	*A. thaliana*	*At*TTM3	2xPi, 3xMn	ExExK, VxRxR, ExE	TTM	Martinez et al (2015)	9
	I1I2P2	*B. distachyon*	*Bd*TTM3		ExExK, AxRxR, ExE	TTM (shown is also low AC)	Swiezawska et al (2020)	9
6YP4	E1AQY1	*H. hybrid cultivar*	*Hp*AC1	GCP, 2xMg, metals not coordinated the usual way, nucleotides from two protomers are stacked	ExExK, AxRxR, ExE	TTM, no measurable AC activity	Kleinboelting et al (2020)	9
2FJT	A0A384LKY8	*Y. pestis*	CyaB	empty	HFxxxxExExK, SxVxR, ExA	AC	Gallagher et al (2006)	10
3N0Y	A0A384LKY8	*Y. pestis*	CyaB	AMPCPP+Mn	HFxxxxExExK, SxVxR, ExA	AC	Gallagher et al (2011)	10
3N0Z	A0A384LKY8	*Y. pestis*	CyaB	3AT+Mn	HFxxxxExExK, SxVxR, ExA	AC	Gallagher et al (2011)	10
3N10	A0A384LKY8	*Y. pestis*	CyaB	cAMP+Mn+Mn	HFxxxxExExK, SxVxR, ExA	AC	Gallagher et al (2011)	10
2ACA	Q87NV8	*V. parahaemolyticus*		2x PO4	HFxxxxExExK, IxRxV, ExA	AC	unpublished	10

Table S2: Data collection and refinement statistics

*Sa*TTM	open state/SO_4	PPPi/Ca^{2+}	PPPi/Mg^{2+}	PPi/Ca^{2+}	ATP/Ca^{2+}	PPi/K^{+}
PDB code	7NS8	7NS9	7NSF	7NSA	7NSD	7OA2
Data collection						
Synchrotron beamline	ESRF ID29	ESRF ID29	ESRF ID23-1	ESRF ID30B	ESRF ID29	ESRF ID23-1
Wavelength (Å)	0.979	0.979	0.972	0.976	0.979	0.972
Resolution range (Å)	44.4-2.3 (2.38-2.30)	46.0-1.75 (1.81-1.75)	43.2-2.0 (2.07-2.0)	46.0-1.95 (2.02-1.95)	45.8-2.2 (2.27-2.19)	41.4-2.7 (2.8-2.7)
Space group	$P4_12_12$	$P4_12_12$	$P4_12_12$	$P4_12_12$	$P4_12_12$	$P4_12_12$
Unit cell (Å, °)	57.66, 57.66, 139.15, 90, 90, 90	60.67, 60.67, 141.09, 90, 90, 90	61.0, 61.03, 136.62, 90, 90, 90	60.55, 60.55, 141.63, 90, 90, 90	60.67, 60.67, 139.35, 90, 90, 90	61.31, 61.31, 138.54, 90, 90, 90
Total reflections	22129 (2148)	51470 (5143)	36416 (3558)	38954 (3885)	28144 (2738)	11928 (1176)
Unique reflections	11068 (1074)	25885 (2581)	18208 (1778)	19769 (1955)	14072 (1367)	6390 (636)
Multiplicity	2.0 (2.0)	2.0 (2.0)	2.0 (2.0)	2.0 (2.0)	2.0 (2.0)	1.9 (1.8)
Completeness (%)	99.95 (100.00)	94.31 (96.81)	98.92 (99.94)	98.87 (99.69)	99.95 (99.78)	78.58 (82.36)
Mean $I/\sigma(I)$	29.77 (15.39)	11.44 (2.12)	17.55 (2.43)	16.60 (2.65)	17.15 (4.12)	8.70 (1.65)
Wilson B-factor (Å^2)	34.52	32.86	43.90	37.37	48.39	58.48
R_{merge}	0.01 (0.04)	0.02 (0.30)	0.01 (0.26)	0.02 (0.27)	0.01 (0.17)	0.03 (0.38)
R_{meas}	0.02 (0.06)	0.03 (0.41)	0.02 (0.37)	0.03 (0.38)	0.02 (0.24)	0.05 (0.54)
CC1/2	1 (0.99)	1 (0.35)	1 (0.88)	1 (0.73)	1 (0.86)	1 (0.78)
Refinement statistics						
Resolution range (Å)	44.4-2.3	46.0-1.75	43.2-2.0	46.0-1.95	45.8-2.2	41.4-2.7
R_{work}/R_{free} (%)	22.6/25.7	18.9/23.5	20.7/23.9	22.5/24.6	22.4/25.3	28.4/31.7
Average B-factor (Å^2)	41.4	51.5	59.9	47.2	63.4	61.29
No. of atoms	1476	1641	1607	1619	1572	1487
macromolecules	1341	1477	1479	1465	1455	1447
ligands	24	26	14	27	37	10
solvent	111	138	114	127	80	30
R.m.s.d., (bonds. Å)	0.010	0.007	0.008	0.018	0.011	0.012
R.m.s.d., (angles, °)	1.15	0.73	1.09	1.93	1.48	1.49
Ramachandran plot						
favoured (%)	97.44	97.16	97.71	97.69	96.55	96.57
allowed (%)	2.56	2.84	2.29	2.31	3.45	3.43
outliers (%)	0.00	0.00	0.00	0.00	0.00	0.00
Rotamer outliers (%)	2.05	0.62	0.62	2.47	0.00	0.65
Clashscore	3.68	1.34	3.70	4.00	3.04	4.52
